# The role of the poly(A) tract in the replication and virulence of tick-borne encephalitis virus

**DOI:** 10.1038/srep39265

**Published:** 2016-12-16

**Authors:** Naveed Asghar, Yi-Ping Lee, Emma Nilsson, Richard Lindqvist, Wessam Melik, Andrea Kröger, Anna K. Överby, Magnus Johansson

**Affiliations:** 1 Södertörn University, School of Natural Science, Technology & Environmental Studies, Huddinge, 14189, Sweden; 2 Örebro University, School of Medical Sciences, Örebro, 70182, Sweden; 3 Örebro University, iRiSC - Inflammatory Response and Infection Susceptibility Centre, Faculty of Medicine and Health, Örebro, 70182, Sweden; 4 Umeå University, Department of Clinical Microbiology, Virology, Umeå, 90185, Sweden; 5 Umeå University, The Laboratory for Molecular Medicine Sweden (MIMS), Umeå, 90185, Sweden; 6 Helmholtz Centre for Infection Research, Innate Immunity and Infection, Braunschweig, 38124, Germany; 7 University of Magdeburg, Institute for Microbiology, Magdeburg, 39120, Germany

## Abstract

The tick-borne encephalitis virus (TBEV) is a flavivirus transmitted to humans, usually via tick bites. The virus causes tick-borne encephalitis (TBE) in humans, and symptoms range from mild flu-like symptoms to severe and long-lasting sequelae, including permanent brain damage. It has been suggested that within the population of viruses transmitted to the mammalian host, quasispecies with neurotropic properties might become dominant in the host resulting in neurological symptoms. We previously demonstrated the existence of TBEV variants with variable poly(A) tracts within a single blood-fed tick. To characterize the role of the poly(A) tract in TBEV replication and virulence, we generated infectious clones of Torö-2003 with the wild-type (A)_3_C(A)_6_ sequence (Torö-6A) or with a modified (A)_3_C(A)_38_ sequence (Torö-38A). Torö-38A replicated poorly compared to Torö-6A in cell culture, but Torö-38A was more virulent than Torö-6A in a mouse model of TBE. Next-generation sequencing of TBEV genomes after passaging in cell culture and/or mouse brain revealed mutations in specific genomic regions and the presence of quasispecies that might contribute to the observed differences in virulence. These data suggest a role for quasispecies development within the poly(A) tract as a virulence determinant for TBEV in mice.

Tick-borne encephalitis virus (TBEV) is an important vector-borne virus causing severe central nervous system disease in humans. Both the prevalence and incidence of the disease have increased in recent decades^1^, and about 10,000 people throughout the world are infected by TBEV annually^2^. TBEV is divided into three genetically distinct subtypes – European- (Eu-), Far Eastern- (FE-), and Siberian- (S-) TBEV^3^. The ~11 kb single-stranded, positive-sense RNA genome of TBEV encodes a single polyprotein flanked by 5′ and 3′ non-coding regions (NCRs). Viral and host proteases process the polyprotein into three structural proteins – capsid (C), precursor membrane (prM), and envelope (E) – and seven non-structural (NS) proteins – NS1, NS2A, NS2B, NS3, NS4A, NS4B, and NS5^3^. Compared to the highly conserved 5′NCR, the 3′NCR can be divided into a conserved and variable region (V 3′NCR)^4^,^5^. The V 3′NCRs of several Eu-TBEV strains contain internal poly(A) sequences that vary in length, whereas the highly virulent Eu-TBEV strain Hypr lacks a large portion of the V 3′NCR, including the poly(A) tract^5^. Tick-borne encephalitis is characterized by a range of symptoms from mild flu-like symptoms to severe encephalitis and paralysis. The severity of disease also differs between Eu-, S-, and FE-TBEV subtypes, with Eu-TBEV resulting in milder disease with 1–2% mortality, while S- and FE-TBEV cause more severe disease with mortality rates of 2–3% and 20–40%, respectively (reviewed in ref. 6). Little is known about the molecular determinants of neuroinvasiveness and neurovirulence of these viruses, but it has been suggested that within the quasispecies pool of TBEV, the virus variants with neurotropic properties become dominant in mammalian hosts resulting in neurological symptoms^7^. We have previously reported the existence of TBEV variants with variable poly(A) lengths within a single blood-fed tick^8^, and we hypothesized that the observed poly(A) heterogeneity was the result of a shift in the TBEV quasispecies pool within the tick in the presence of mammalian blood at 37 °C.

The Eu-TBEV strain Neudoerfl has the longest known TBEV genome, and it contains a 30–250 nt poly(A) tract^5^. The V 3′NCR of Neudoerfl is dispensable for virus replication in cell culture and for virulence in mice^9^, but in ticks the V 3′NCR was predicted to play an important role in the viral life cycle^5^,^10^. Recent studies with FE-TBEV have shown that the V 3′NCR and poly(A) insertion are important virulence determinants^11^,^12^, but the underlying pathophysiological mechanism behind this is unclear.

Evidence for the effect of poly(A) tract length on virulence and the development of TBEV quasispecies in different hosts is missing. Infectious cDNA clones can be used to generate viral stocks with defined genetic backgrounds^13^, and this makes them the perfect tool for performing such studies. Here we report the generation of two infectious clones – Torö-6A and Torö-38A – with short and long poly(A) tracts, respectively. Characterization of the rescued viruses revealed substantial differences in their replication kinetics in cell culture and in their virulence in mice. Viruses derived from these infectious clones were passaged in cell culture or mice followed by sequencing with next generation sequencing (NGS) and Sanger sequencing to understand the genetic determinants that are important for virus replication in diverse cellular environments. We found that the length of the poly(A) tract is important in determining the variability of the genetic pool of TBEV because the virus with a short poly(A) tract was genetically more stable compared to the virus with a long poly(A) tract. NGS analysis also identified genomic elements favoured by replication in cell culture and mouse, revealing host-specific genetic divergence.

## Results

### Introduction of a long poly(A) in Torö-2003 attenuates virus replication in cell culture

**Figure 1:**
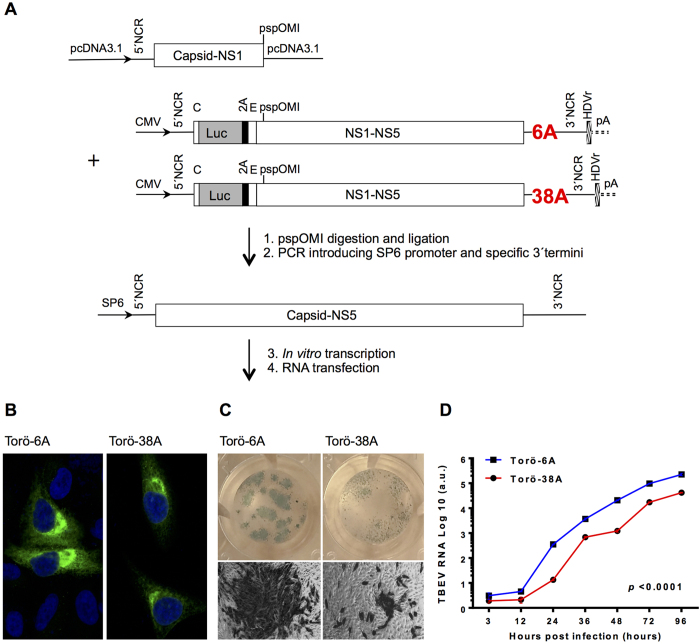
Characterization of infectious clone *in vitro.* (**A**) Schematic view of the strategy used to rescue Torö-6A and Torö-38A infectious clones. A DNA fragment containing the 5′ NCR, the structural genes, and 124 bp of NS1 was excised from its clone in the pcDNA3.1 vector. Another fragment comprising the non-structural proteins (NS1–5) and the 3′ NCR containing 6 or 38 adenine nucleotides was excised from Torö-2003 luciferase sub genomic replicons. Both fragments were ligated at the pspOMI restriction site, and PCR was used to introduce specific 3′ termini and a SP6 promoter preceding the 5′ NCR. A mixture of HEK293 and Vero B4 cells was transfected with the *in vitro*-transcribed RNA to rescue the infectious clones. (**B**) Immunofluorescence assay of Torö-6A or Torö-38A–infected A549 cells using primary antibody against the E protein and Alexa 488 as the secondary antibody. **(C)** Plaque morphology of Torö-6A or Torö-38A in Vero B4 cells. **(D)** Vero B4 cells (n = 3) were infected with Torö-6A and Torö-38A at an MOI of 0.1, and viral RNA levels were quantified by real-time RT-PCR at the indicated time points. The viral RNA was standardized to the mRNA levels of GAPDH and presented as the mean ± SEM. Statistical significance calculated by ordinary 2-way ANOVA *P* < 0.0001. a.u., arbitrary unit.

Torö-2003 was originally sequenced directly from field-caught *I. ricinus* ticks without conventional enrichment procedures such as propagation in cell culture and/or suckling mouse brain, and it contains a short poly(A) tract, (A)_3_C(A)_6_^10^,^14^. To determine how the length of the poly(A) tract impacts Eu-TBEV replication and pathogenicity, we constructed an infectious clone based on Torö-2003 (GenBank Accession no. DQ401140.2) and compared its replication with a variant clone, where a longer poly(A) tract, (A)_3_C(A)_38_, was introduced. The (A)_3_C(A)_38_ sequence is the longest poly(A) tract yet detected in TBEV in nature and was sequenced directly from a blood-fed tick^8^. Viral RNA was generated from the infectious clones Torö-6A and Torö-38A (Fig. 1A) by *in vitro* transcription, and the viruses were recovered from supernatants of RNA-transfected HEK-293 and Vero B4 cells (ratio 1:1). HEK-293 and Vero B4 cells provided transfection and replication advantages, respectively.

Different methods were used to characterize the recovered viruses. First, immunofluorescence staining of E protein of TBEV was performed to confirm the viability of the released rescued viruses (Fig. 1B). Second, the plaque morphology was assessed and found to be different between the two strains. Whereas Torö-6A showed large plaques similar to Hypr (data not shown), the morphology of Torö-38A plaques was small and diffuse (Fig. 1C). Third, the replication kinetics of the rescued viruses were compared in Vero B4 cells following infection with Torö-6A or Torö-38A at a multiplicity of infection (MOI) of 0.1 by measuring viral RNA level using real time RT-PCR. Torö-6A replicated significantly faster than Torö-38A (Fig. 1D, 2-way ANOVA, P < 0.0001). Taken together, these results demonstrated the successful recovery of both infectious clones and revealed distinct phenological differences between them.

### Torö-38A is more pathogenic than Torö-6A in mice

**Figure 2:**
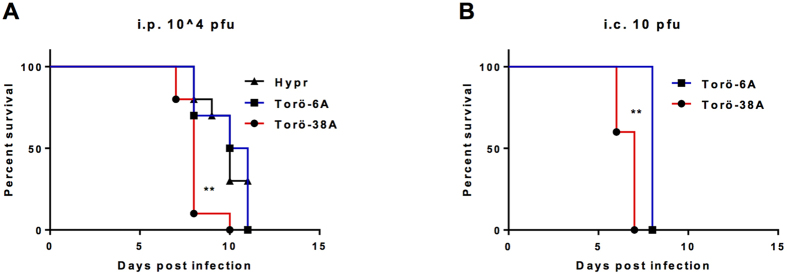
Pathogenicity of Torö-6A and Torö-38A *in vivo*. C57BL/6 mice (n = 10) were inoculated **(A)** intraperitoneally (10^4^ pfu/mouse) or **(B)** intracranially (10 pfu/mouse) with the indicated virus. The survival rate was then monitored. Survival differences were tested for statistical significance with the log-rank (Mantel–Cox) test, **p < 0.01.

We have previously demonstrated that intraperitoneal (i.p.) inoculation of the TBEV strain Hypr causes mortality in adult wild-type mice^15^,^16^. We were therefore interested in comparing the virulence of these new Swedish strains rescued from infectious clones with the highly pathogenic Hypr strain in mice. Interestingly, i.p. inoculation (10^4^ pfu) of Torö-38A resulted in a mean survival time of 8 days, whereas mice infected with Torö-6A or Hypr survived significantly longer at 10 days (Fig. 2A, log-rank (Mantel-Cox) test, **p < 0.01). Next, the neurovirulence of Torö-6A and Torö-38A was compared. Adult mice were intracranially (i.c.) inoculated with 10 pfu of virus, and survival time was monitored. Torö-6A–infected mice died on day 8 post infection (p.i.), whereas Torö-38A showed stronger neurovirulence, and mice succumbed as early as day 6 to 7 p.i. (Fig. 2B, log-rank (Mantel–Cox) test, **p < 0.01). Together, these results showed that Torö-38A was both more pathogenic and more neurovirulent in C57BL/6 mice compared to Torö-6A.

### The role of the poly(A) tract in quasispecies emergence in mice

**Figure 3:**
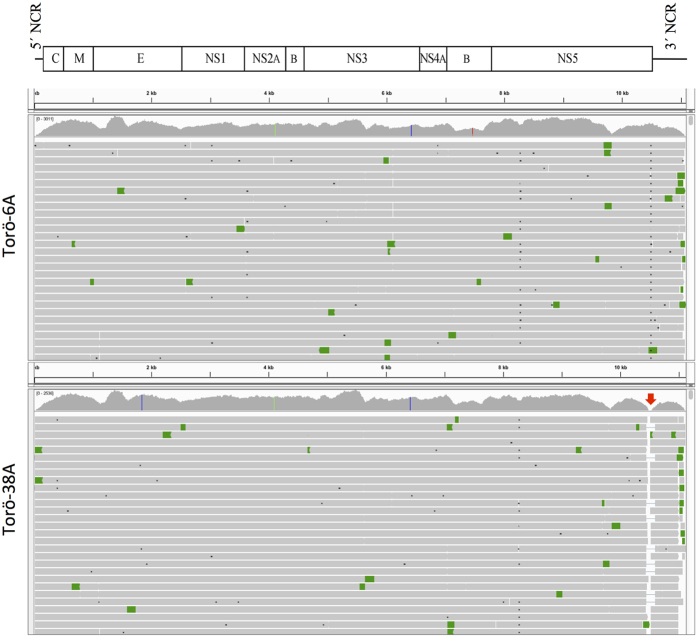
Integrative Genomics Viewer alignment of next generation sequencing reads of Torö-6A and Torö-38A isolated from mouse brains. Schematic view of the complete genome of Torö-2003 is illustrated above the alignment. The vertical coloured lines in the coverage plots correspond to nucleotides that differ compared to the reference genome. The alignment is coloured by pair orientation where grey horizontal bars are properly aligned read pairs and green bars are read pairs that differ from the expected orientation. The poor coverage across the poly(A) tract of Torö-38A is indicated by a red arrow.

To examine how the poly(A) tract affects quasispecies development in the virus population, the genetic variations of the two different Torö strains were determined after the infection in mice. Three mice were inoculated i.p. with Torö-38A or Torö-6A and sacrificed on day 7 or 8 p.i., respectively, when clinical symptoms appeared. Viral RNA from the brains was analysed by NGS. The inoculated input viruses (P0) were also included in the NGS analysis. An average depth of 1,548 and 1,394 reads per position was obtained for Torö-6A and Torö-38A, respectively. NGS coverage for the Torö-6A genome was fairly uniform, but it was very shallow across the poly(A) region of Torö-38A (Fig. 3). Because the length of the poly(A) tract was the only difference between the rescued viruses, we initially investigated the poly(A) region of both viruses. NGS revealed variability in the length of poly(A) tract and deletions within the V 3′NCR of the recovered viruses. Torö-6A showed single nucleotide (A or G) deletions, whereas Torö-38A variants demonstrated larger deletions (Table 1).

**Figure 4:**
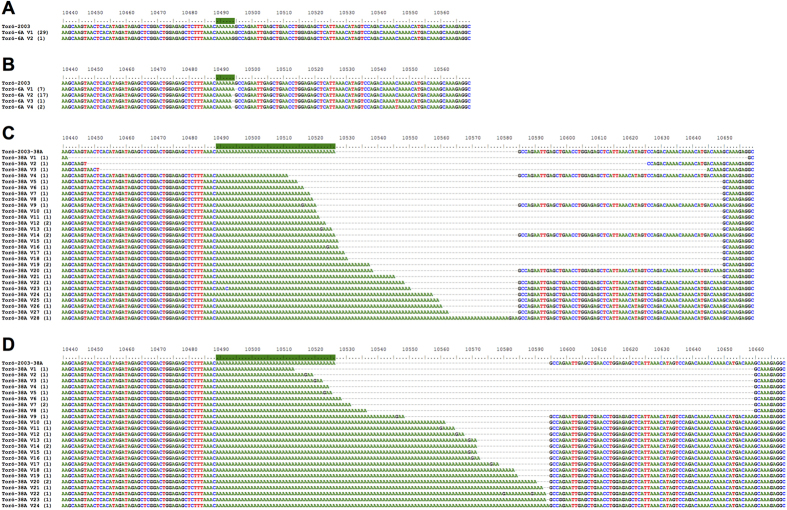
Alignment of 3′ NCR partial sequences from pcDNA3.1 clones of the rescued viruses. The sequences correspond to Torö-6A and Torö-38A from cell culture **(A**,**C)** and mouse brain **(B**,**D)**. The number of sequenced clones with identical sequences is shown in parentheses. Nucleotide number 10440 corresponds to the TBEV strain Torö-2003. The poly(A) tract in the reference sequence is highlighted by a green line.

The resolution of long stretches of homopolymeric nucleotide sequences, like poly(A) tracts, is low using modern sequencing techniques like NGS^17^. To overcome this issue, we cloned PCR products of the NS5–3′NCR of rescued viruses. Approximately 30 random clones of each virus from cell culture (P0) and mouse brain were individually sequenced by conventional techniques. The poly(A) tract of Torö-6A from P0 was quite stable, with 29 out of 30 sequenced clones showing a wild-type poly(A) tract sequence (Fig. 4A). However, none of the sequenced clones from mice showed wild-type poly(A) tracts (Fig. 4B). In the case of Torö-38A, Sanger sequencing of clones from P0 and mice showed great variability within their V 3′NCRs, and about 90% of the sequenced clones were unique variants (Fig. 4C,D), thus supporting the NGS data. We observed both large deletions across the poly(A) tract as well as increasing lengths of the poly(A) tract. A longer poly(A) tract seemed more common in viruses sequenced from mice compared to cell culture (Fig. 4C,D). The initial cDNA-derived virus should theoretically generate a single variant of TBEV identical to the parental clone. However, both the NGS and Sanger sequencing analysis revealed that viral variants developed rapidly within the TBEV pool and were already present in the viruses recovered at P0. The more virulent Torö-38A showed a greater diversity in the number of quasispecies compared to Torö-6A (Table 1, Fig. 4).

### TBEV quasispecies arise through purifying selection and the generation of new mutations

Torö-38A from P0 and mice showed high variability in the length of the poly(A) tract and deletions in the V 3′NCR, indicating instability of the virus with a 38A poly(A) tract in both cell-culture and *in vivo* mammalian environments. In contrast, Torö-6A from P0 appeared to be more stable than Torö-6A from mice (Fig. 4). The Vero B4 cells might have had an effect on quasispecies development because it is a very susceptible cell line and lack parts of the innate immune response; therefore, we performed passaging experiments using immunocompetent A549 cells. To further study the evolution and stability of Torö-6A under cell culture conditions and to compare this with the Torö-6A virus that evolved in mice, the virus was passaged five times (P5) in A549 cells followed by NGS. To identify single nucleotide polymorphism (SNPs) and to differentiate them from the nucleotide changes introduced in the infectious clones during the rescue process, we aligned the NGS data against the Torö-2003 sequence (GenBank Accession no. DQ401140.2). With the exception of a single silent mutation at position 6423, we could not identify any nucleotide changes incorporated during the rescue process, indicating the high fidelity of the polymerases used. Passaging the virus in A549 cells resulted in a mutation in the E protein of Torö-6A that increased the net positive charge of the virus surface (Table 2). SNP analysis supported the existence of quasispecies within the TBEV pools of rescued viruses from P0, P5, and mouse brain. In addition, the quasispecies exhibited host-specific genetic divergence (Table 2) that might be a consequence of the selection pressure encountered in cell culturing and/or animal hosts. Most of the dominant SNPs in P5 and mouse TBEV pools of Torö-6A and Torö-38A clearly arose from already existing quasispecies variants present in P0. However, a valine to leucine change at 7462 was only observed in the Torö-6A pool from mice, indicating that these variants were generated by a mutation occurring in the mouse rather than through the purifying selection of already existing variants in P0. A number of SNPs were only observed in one of the three Torö-6A or Torö-38A isolates from mice and cell culture (Supplementary Table S1). In line with previous work^9^,^18^, truncations in Torö-6A were observed after passaging in A549 cells (data not shown). These results suggest that selection of already existing variants is not the only factor that governs quasispecies development, and specific mutations generated after the introduction of the virus into particular environments might also occur.

### Genomic stability of rescued viruses

**Figure 5:**
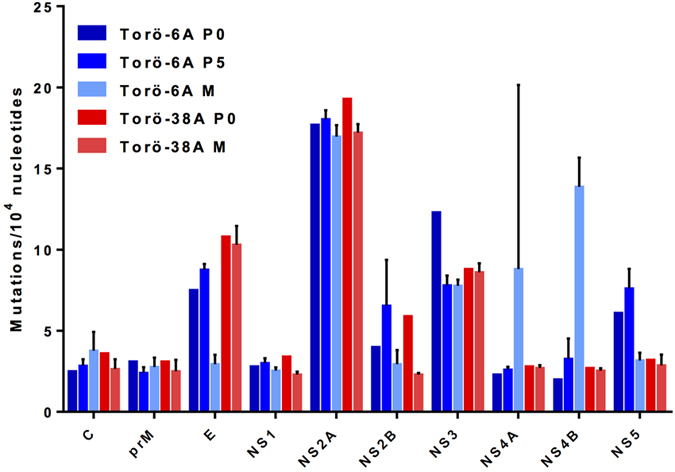
Genomic diversity across the open reading frames of Torö-6A and Torö-38A from cell culture and mice. For each gene, the number of total mutations was divided by the number of total nucleotides sequenced. Mutation frequencies are presented as mutations per 10,000 nucleotides. Error bars represent the standard error of the mean.

The genomic stability of RNA viruses is dependent on the selection pressure present in different hosts. Entire genome or open reading frame (ORF) sequencing is a requisite to map all the genes involved in the expressed phenotype of a virus^13^. The overall mutation frequency of the ORF was almost identical for all viruses from P0, P5, and mouse brains (data not shown), but noticeable differences in mutation frequencies of individual genes were observed. In general, the NS2A gene of all of the viruses showed the highest number of mutation frequencies at 16.3–19.3 mutations/10,000 nt. The C, prM, and NS1 genes of the rescued viruses appeared to be the most stable genes in both cell culture and mice (Fig. 5). Interestingly, the E gene of Torö-6A was more stable in mice than in cell culture whereas the NS4B gene had higher mutation frequencies in mice (Fig. 5). The opposite trend was observed in Torö-38A in which higher mutation frequencies were seen in E while NS4B was quite stable (Fig. 5). Despite low replication in cell culture, Torö-38A was highly pathogenic in mice (Figs 1 and 2). Because the mutation frequency of every gene of Torö-38A except NS2B was similar in cell culture and in mice, the observed replication versus virulence differences might be due to specific interactions involving NS2B.

### Predicting the role of the 3′NCR in TBEV pathogenicity

**Figure 6:**
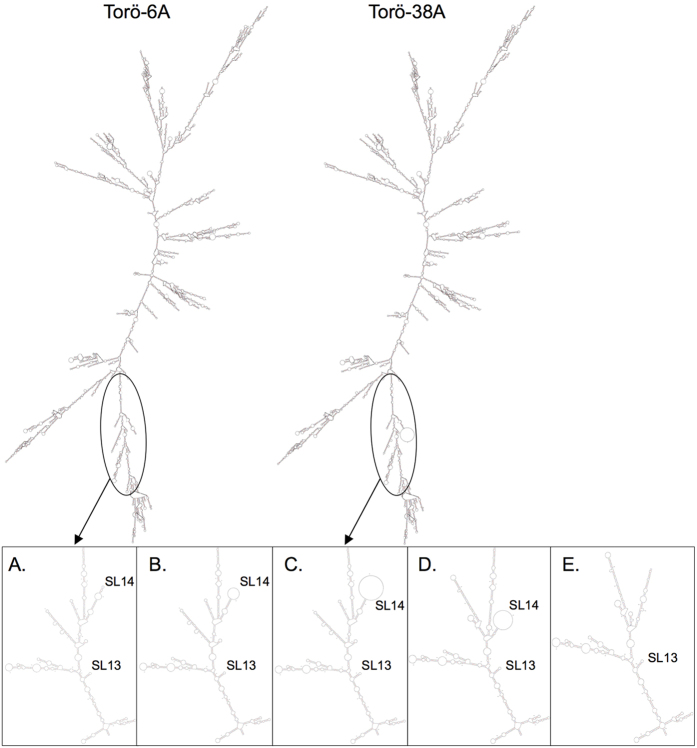
RNA secondary structure prediction of the Torö genomes. The upper panel illustrates RNA folding of Torö-6A and Torö-38A sequences at 37 °C using mfold (Zuker 2003). The lower panel illustrates folding of the 706 proximal and 701/733 terminal bases to enhance the resolution of the secondary structures in the 3′ and 5′NCRs. **(A,B)** Present folding of Torö-6A and the variant with a guanine deletion at nucleotide 10495, respectively. Folding of Torö-38A **(C)**, the nucleotides 10527–10592 deletion **(D),** and the nucleotides 10442–10599 deletion **(E)** are also depicted. RNA folding predictions show that stem loop (SL) 14 is the only RNA secondary structure affected by the observed deletions and the poly(A) sequence of Torö-38A.

The 3′NCR of RNA viruses forms specific RNA secondary structures, especially stem loops (SLs), that are important for virus replication and viability. The 3′NCR of Torö-2003 has been shown to form 14 SLs where SL1–5 constitute the promoter region and SL6–14 are present within the enhancer region of the 3′NCR^4^,^14^. RNA folding of Torö-6A and Torö-38A sequences revealed that SL14 was the only RNA secondary structure affected by the increased poly(A) length of Torö-38A (Fig. 6A,C). An adenine deletion at nucleotide 10489 of Torö-6A did not interrupt SL14, whereas a guanine deletion at nucleotide 10495 resulted in disruption of SL14 (Fig. 6B). The latter deletion was observed in 71% of the TBEV variants from one Torö-6A–infected mouse, and this mouse showed the most severe pathological symptoms (data not shown). The two major deletions observed in the 3′NCR of Torö-38A (Table 1) also affected SL14. The deletion at nucleotides 10527–10592 resulted in disruption of SL14 (Fig. 6D) and the deletion at nucleotides 10442–10599 completely eliminated SL14 (Fig. 6E). These results indicated that the major genomic differences between Torö-6A and Torö-38A were localized to the V 3′NCR and seemed to affect the formation of SL14.

## Discussion

Variations in the 3′NCR of tick-borne flaviviruses can be determinants of virulence^11^,^12^. In previous work we demonstrated the existence of variable 3′NCRs in nature from TBEV strains amplified directly from ticks^8^. However, the biological role of these specific variations is not clear. In this study we rescued two TBEV infectious clones, Torö-6A containing the wild type (A)_3_C(A)_6_ Torö-2003 poly(A) tract and Torö-38A with a modified (A)_3_C(A)_38_ poly(A) tract. In line with Sakai *et al*., the virus with the longer poly(A) tract was more pathogenic in mice, indicating a putative pathophysiological role of the longer poly(A) tracts that have been detected in Eu-TBEV variants. However, contrary to the previous studies that demonstrated inviolate TBEV replication after poly(A) insertion^11^,^19^, poly(A) insertion in the V 3′NCR of Torö-2003 restricted viral replication in cell culture. The observed replication discrepancies could be due to the different TBEV subtypes or to higher stability of the replicon RNA used in the respective studies^11^,^19^.

It was recently established that the V 3′NCR and the poly(A) tract modulate the virulence of FE-TBEV^11^,^12^. In contrast, previous work has suggested that the V 3′NCR is dispensable for virulence of Eu-TBEV because removal of the complete V 3′NCR had no effect on TBEV virulence^9^. Mandl *et al*. also observed truncations in the variable 3′NCR leading to complete removal of the poly(A) tract after 15–20 passages of the virus or the infectious clone. Here we show that a long poly(A) tract enhances the neuroinvasiveness and neurovirulence of Eu-TBEV strain Torö-2003 immediately after virus rescue. This might be related to the observed rapid development of quasispecies because rescued Torö-38A contained virus variants with variable poly(A) lengths and different partial deletions within the V 3′NCR already at P0. In addition, Torö-38A was significantly more virulent than the Eu-TBEV strain Hypr, which lacks a poly(A) tract. Extensive investigation of the quasispecies populations showed that no particular length of the poly(A) tract was favoured during virus replication in cell culture or in the brains of infected mice (data not shown).

Quasispecies with high genomic diversity have been shown to have better adaptability to changing host environments^20^. For example, the neurotropism and pathogencity of poliovirus has been coupled to high quasispecies diversity^20^, and Asibi, a neurotropic yellow fever virus (YFV) strain, contains a more diverse quasispecies population compared to the attenuated progeny YFV vaccine strain (17D)^21^. The overt quasispecies diversity of Torö-38A compared to Torö-6A is a factor that might underlie the observed differences in neuroinvasiveness and neurovirulence. Hepatitis C virus (HCV) is another member of the family *Flaviviridae*, but it contains a poly(U) tract (U-core). Differences in length of the U-core remain the strongest determining factor in RIG-I–dependent induction and might be responsible for the clearance of acute infection^22^. However, because the rescued TBEVs with longer poly(A) tracts exhibited a highly pathogenic phenotype, this might indicate an increased capacity to evade viral sensing and type I interferon responses. A putative mechanism for such pathogenicity might be the lower replication rate of Torö-38A that facilitates efficient hiding of viral double-stranded RNA in the intracellular membrane vesicles^23^. Or it could be that the poly(A) tract of Torö-38A contributes to the observed virulence by forming or stabilizing the RNA secondary structures within the 3′NCR that are essential for the generation of subgenomic flavivirus RNA, a critical virulence determinant in flavivirus infections^24^,^25^,^26^,^27^.

Besides TBEV, internal poly(A) or poly(U) tracts have been described in a number of different viruses^28^,^29^,^30^,^31^,^32^,^33^. The negative-sense, vesicular stomatitis virus (VSV) generates a poly(A) tail through an RNA dependent RNA polymerase (RdRp) slippage mechanisms, which depends on specific sequences and a U7 tract on the RNA template^34^. The positive-sense, chikungunya virus (CHIKV) strain S27 was found to contain an internal poly(A) sequence that varies from 19A to 106A nucleotides and a template-dependent slippage mechanism for poly(A) variability, similar to VSV was suggested^32^. Interestingly, in other CHIKV strains the long variable poly(A) is replaced by a 4A or 6A sequence at the homologous position^32^,^35^, which is similar to the poly(A) tract normally identified in Eu-TBEV strains, e.g. Ljubljana and Torö-2003^5^,^10^. Khan *et al*. proposed that additional U nucleotides might have been added while cell culturing to generate an U7 sequence, critical for polyadenylation through RdRp slippage. Because the 3′NCR of Torö-6A is much more stable than Torö-38A a similar mechanism of rapid poly(A) elongation through RdRp slippage could be suggested. However, as several A6 sequences are found within the Eu-TBEV strains and extension in poly(A) sequence occurs at a specific site, additional experiments are required to further characterise specific sequences that regulates such a mechanism in positive RNA viruses. In addition, elongation of the viral poly(A) tracts has been observed in ticks and mosquito cells^8^,^32^.

The error-prone polymerase of RNA viruses is responsible for generating the diversity of viral quasispecies that facilitates virus evolution and provides adaptive fitness advantages in diverse environments^36^,^37^. TBEV is known to exist as clusters of quasispecies within ticks and mammals^7^,^8^,^38^,^39^. Quasispecies populations are influenced by the host environment, and changes in the population might affect the phenotype of specific strains^40^. In addition, studies have shown that specific quasispecies populations evolve when flaviviruses are rescued from cDNA clones^9^,^41^. A cDNA clone of the Neudoerfl strain with a (A)_3_C(A)_49_ poly(A) tract insertion was previously shown to accumulate similar partial deletions within the V 3′NCR in cell culture and in mice^9^. Together with our findings using the (A)_3_C(A)_38_ insertion, these observations suggest that a long poly(A) tract induces instability within the TBEV genome that might favour evolution and the adaptation of quasispecies populations when the virus switches from ectothermic/tick to endothermic/mammalian conditions.

In previous studies, several mutations in the E gene, all of which increased the net positive charge on the virus surface, were characterized within domain (D) I–III of the E protein^42^,^43^,^44^,^45^,^46^,^47^,^48^,^49^. These mutations enhance virus entry into certain cell types by increasing the binding affinity to heparan sulfate receptors^50^. However, neuroinvasiveness has been shown to decrease because these mutations impair virus spreading, leading to low viremia and a consequent failure to invade the CNS^50^. The rescue of Torö-6A in Vero B4 cells (P0) generated TBEV variants with mutations in the E protein that all increased the net positive charge on the virus surface (Table 2). These mutations – DI^178,Glu→Lys^, DII^236,Asn→Lys^, and DIII^320,Asp→Asn^ – were not detected in the previous studies^42^,^43^,^44^,^45^,^46^,^47^,^48^,^49^ but support the increased net positive charge seen in TBEV after cell culture. Interestingly, two of these mutations disappeared after passaging in A549 cells, and only the DII^236,Asn→Lys^ mutation remained as a dominant variant (100%) at P5. After infection in mice, all three mutations had reverted, providing further evidence that the net positive charge switch is an *in vitro* phenomenon occurring primarily in cell culture. For the E protein of Torö-38A, no selection of positively charged residues was detected at P(0). Because an increasingly positive charge decreases virulence^50^, this might be an additional factor explaining the differences in virulence observed here.

Several additional quasispecies within the TBEV pools of passaged viruses were found in the SNP analysis. The SNPs that appeared in the P0 population and subsequently became dominant in P5 and/or mice (Table 2) might be a consequence of selection pressures encountered in cell culture and/or an animal host. Besides selected and reverted SNPs from P0, we observed an SNP generated by a mutation in NS4B of Torö-6A isolates from mouse brain. Our data suggest that virus adaptation to a new environment occurs not only by selection of existing variants within the quasispecies population^7^, but also by emergence of new random mutations.

We evaluated population diversity within each isolated virus by calculating mutation frequencies for the complete ORF as described previously^13^. We did not observe any noticeable difference in total mutation frequencies calculated over the ORF of Torö-6A and Torö-38A despite the significant replication differences between the two viruses (*p* < 0.05), indicating that fidelity of the RNA virus polymerase is not critically linked to replication kinetics^13^. Although total mutation frequencies for the ORFs of these viruses were similar, noticeable differences in mutation frequencies of individual genes were observed both between the two viruses and among different isolates of the same virus. It seems that there exists a balance in the overall mutation frequencies within the ORF, where higher mutations in one gene might be compensated for by lower mutations in another gene. These findings strongly encourage the use of the complete ORF rather than a particular gene to compare mutation frequencies among different viruses.

In our previous work, fourteen SL structures were predicted for the 3′NCR of Torö-2003^14^. RNA folding predictions of the Torö-6A and Torö-38A genomes and their V 3′NCR variants showed that SL14 was the only secondary structure affected by poly(A) insertions or deletions within the V 3′NCR. We have previously shown that the Neudoerfl strain in our lab contains two distinct quasispecies variants with deletions in their V 3′NCRs^8^. RNA folding predictions of these variants revealed that this strain also lacks SL14 (data not shown). Our current data suggest that the poly(A) insertion within the V 3′NCR of Torö-2003 restricts the virus replication rate in cell culture but enhances neuroinvasiveness and neurovirulence. One possible explanation for the enhanced neuropathogenicity could be that the intact SL14 conformation is needed to interact with components of the host immune system. Several host proteins, including EF-1α, FBP1, La, Mov34, and p100, have been reported to interact with the 3′NCR of flaviviruses^51^,^52^,^53^,^54^,^55^,^56^. However, further studies are required to identify cellular interacting partners and to determine if the interactions are dependent on binding to SL14.

## Methods

### Cells and viruses

Simian Vero B4 cells, human embryonic kidney (HEK293) cells, and human lung carcinoma A549 cells (American Type Culture Collection) were maintained in Dulbeccos’s modified Eagle’s medium (DMEM; Hyclone) containing 5% fetal calf serum (FCS) with penicillin and streptomycin (Life Technologies).

Two infectious clones, Torö-6A and Torö-38A, were rescued. The infectious clones were constructed from two plasmids (pCDNA3.1-TBEV-CME and pTBEV-*luc-*rep) (Fig. 1A). pCDNA3.1-TBEV-CME comprises the 5′NCR, the structural genes, and 124 bp of NS1 cloned into the pcDNA3.1 vector, and pTBEV-*luc-*rep is a previously established TBEV replicon model for Torö-2003^57^,^58^. For Torö-38A, 32 additional adenine bases were incorporated within the poly(A) tract (nucleotides 10489–10495) of pTBEV-*luc-*rep by an overlapping PCR technique. All of the cloning work was performed using standard molecular biology procedures, and each cloning step was confirmed by sequencing (Eurofins MWG Operon, Ebersberg, Germany). Primer sequences are available upon request. Both segments were linearized and ligated (pspOMI).

A PCR was performed to introduce the SP6 promoter sequence preceding the 5′NCR using the forward primer 5′-AGTCATTTAGGTGACACTATAGAGATTTTCTTGCACGTGC-3′ and the reverse primer 5′-AGCGGGTGTTTTTCCGAGTCACAC-3′. The additional G (underlined) was introduced between the SP6 and 5′NCR sequences to allow for efficient transcription. *In vitro* transcription was performed as per the manufacturer’s instructions using the MEGAscript^®^ SP6 Kit (Ambion), and the cap analog m^7^G(5′)ppp(5′)G (Ambion) was used to synthesize capped RNA. *In vitro*-transcribed RNA was transfected into a mixed (1:1) HEK293 and Vero B4 cell population using Lipofectamine 2000 (Invitrogen). After 96 h, the supernatant from the transfected cells was transferred to Vero B4 cells in T25 flasks. Cells were monitored for the appearance of cytopathic effects at which time the virus was harvested.

### Immunofluorescence

The first passage of rescued viruses was used to infect A549 cells for an immunofluorescence assay. A total of 4 × 10^4^ cells grown on coverslips in 24-well plates were incubated with TBEV Torö-6A or Torö-38A at a MOI of 1 for 1 h at 37 °C in 100 μl serum-free DMEM. The virus inoculum was removed, cells were washed with PBS, 1 ml of DMEM-2% FCS was added, and the incubation was continued at 37 °C for 24 h. Cells were fixed with 3% paraformaldehyde (PFA), quenched with 10 mM glycine, and permeabilized with PBS and 0.5% Triton X-100 followed by incubation with primary antibodies against the E protein (mouse monoclonal 1786,3, diluted 1:500^59^ and secondary Alexa 488 donkey anti-mouse IgG antibodies (Invitrogen, diluted 1:250)). Cells were incubated with DAPI (diluted 1:5000) before the coverslips were mounted and analysed using a Nikon A1R Laser Scanning Confocal Microscope (Nikon) with a 60 × oil immersion lens (Plan Apochromat VC) and NIS-Elements microscope-imaging software (Nikon).

### Plaque assay

Plaque assays were performed as previously described^23^. Briefly, 1 × 10^4^ low-passage Vero B4 cells grown in 96-well plates were incubated with TBEV Torö-6A or Torö-38A in 10-fold dilutions for 1 h at 37 °C in 50 μl DMEM-2% FCS. The inoculum was removed and 100 μl Avicel media (10 ml 2 × DMEM, 10 ml 2.4% Avicel (sterile), 100 μl BSA 10% (sterile)) was added to each well, and incubation was continued for 96 h at 37 °C. Cells were fixed with 3% PFA and stained with primary antibodies against the E protein (mouse monoclonal 1786,3, diluted 1:500^59^ and secondary HRP-conjugated goat anti-mouse IgG (Thermo Fisher Scientific, diluted 1:2000)). Plaques were visualized by adding TrueBlue peroxidase (KPL).

### Viral infection and passaging of Torö-6A and Torö-38A

For replication kinetics and virus passaging, 3 × 10^5^ Vero B4 and 1 × 10^5^ A549 cells were grown in 12-well and 6-well plates, respectively. The cells were infected with Torö-6A or Torö-38A at a MOI of 0.1. As the plaque morphology was different between the two strains the pfu to RNA ration was determined and found to be the same. The infection was terminated at the indicated time points, and the amounts of viral RNA were determined with real-time RT-PCR. For passaging, 48 h p.i. media containing viruses was collected and analysed by plaque assay before the next passage was performed.

### RNA extraction and real-time RT-PCR

Total cellular RNA was extracted using the Nucleospin RNA kit (Macherey-Nagel) according to the manufacturer’s instructions, and 500 ng RNA was used to synthesize cDNA with the QuantiTect reverse transcription kit (Qiagen). mRNA levels of simian GAPDH and human beta actin were measured with validated PrimerPCR (BIO-RAD) and QuantiTect primers (Qiagen), respectively, and the KAPA SYBR FAST qPCR kit using the 7900HT Fast-Real-Time PCR System (Applied Biosystems). Viral RNAs were detected by using previously described specific primer pairs/probes with a TaqMan system against the 3′NCR region of TBEV^60^ and the KAPA PROBE FAST qPCR kit. Values for viral RNAs were normalized to the mRNA values of the two housekeeping genes.

### Ethics statement

All animal experiments were performed in compliance with the German Animal Welfare Law (TierSchG BGBl. S. 1105; 25.05.1998). The mice were housed and handled in accordance with good animal practice as defined by FELASA. All animal experiments were approved by the responsible state office (Lower Saxony State Office of Consumer Protection and Food Safety) under permit number AZ 33.9-42502-04-11/0528.

### Mouse experiments

Six to 8-week old C57BL/6 mice were purchased from Envigo. All animals were housed under pathogen-free conditions at the laboratory animal facility of the Helmholtz Centre for Infection Research. Experiments with TBEV infection were performed in the BSL-3 facility at Helmholtz Centre for Infection Research. Mice were anesthetized by i.p. injection with a mixture of ketamine (100 μg/g body weight) and xylazine (5 μg/g body weight). After anaesthesia, mice were inoculated i.p. (10^4^ pfu/mouse) or i.c. (10 pfu/mouse) with TBEV strain Hypr, Torö-6A, or Torö-38A. The RNA/pfu ratio was the same for all three viruses. For genomic RNA analysis of the infectious clones Torö-6A and Torö-38A, i.p.-inoculated mice were euthanized at 8 and 7 days p.i., respectively. Mice were perfused with 10 ml of PBS, and brain tissues were removed. Each brain was homogenized in TRIzol reagent (Invitrogen) using Lysis matrix (Nordic Biolabs) and a FastPrep-24 the tissue homogenizer (MP) for further genetic analysis.

### NGS

For genomic sequencing, cDNA was synthesized using total RNA isolated from virus-infected Vero B4 cells, A549 cells, and mouse brains as described above. To achieve whole genome coverage, six or seven overlapping fragments were amplified by nested PCR using KOD Hot Start Master Mix (Novagen^®^) as previously described^8^. The PCR products were gel purified using the Wizard^®^ SV Gel and PCR Clean-Up System (Promega), and all fragments for each sample were pooled in a 1:1 molar ratio. The Qubit^®^ dsDNA BR Assay Kit (Life Technologies) was used to determine the concentration of each pooled sample, and stepwise dilutions were made using 10 mM Tris pH 8.5 to obtain a final concentration of 0.15 ng/μl. The Nextera^®^ XT DNA library preparation kit (Illumina^®^) was used to prepare an indexed paired-end multiplexed sequencing library. Tagmentation of 0.75 ng input DNA was performed at 55 °C for 6.5 min followed by PCR amplification as per the manufacturer’s instructions using standard index primers (Illumina^®^). Indexed PCR amplicons were cleaned up using Agencourt AMPure XP beads (Beckman Coulter). The concentration and size distribution of each library were measured with the Qubit^®^ dsDNA BR Assay Kit and the Agilent High Sensitivity DNA kit (Agilent Technologies), respectively, and these values were used to calculate the normality of each library with the formula: χ = (concentration × 10^6^)/(656.6 × average size). Libraries were pooled after normalization and denatured with 0.2N NaOH before loading onto a MiSeq cartridge v2, 300 cycles (Illumina^®^). NGS was performed with a MiSeq desktop sequencer (Illumina^®^) using index reads = 2 and paired-end settings.

### NGS data analysis

The quality of the NGS data was assessed by generating quality-control statistics with FastQC (http://www.bioinformatics.bbsrc.ac.uk/projects/fastqc). Trimmed reads with average quality scores of >30 were aligned to the TBEV genome (NC_001672.1) using TopHat2^61^, and the alignment was visualized with the Integrative Genomics Viewer software^62^. After removal of PCR duplicates (Picard tools, http://picard.sourceforge.net) and file conversion (samtools^63^), base counts were generated with the UnifiedGenotyper from the GATK package^64^. To calculate the mutation frequency for each gene, the total number of nucleotides sequenced was counted. Because different positions had different coverage, the sum of the mutation frequency per position was divided by the total number of nucleotides sequenced. Mutation frequencies are presented as the number of mutations per 10,000 nucleotides.

### 3′ NCR analyses

The NS5–3′NCR fragments of Torö-6A and Torö-38A infectious clones from cell culture and mice were amplified by nested PCR using previously described primer sets^8^ and cloned into pcDNA3.1/V5-His-TOPO^®^ (Invitrogen). For each virus, approximately 30 clones were individually sequenced (Eurofins MWG Operon) and nucleotide sequences were aligned using BioEdit (version 7.1.3.0, Tom Hall Ibis Therapeutics, Carlsbad, CA). Chromatograms of Torö-38A variants with 49A and 57A long poly(A) tracts sequenced in both directions are supplied (Supplementary Data 1–4).

### RNA folding

The folding of the Torö-6A and Torö-38A sequences into secondary RNA structures was predicted using mfold by Zuker and Turner. RNA folding calculations were carried out at 37 °C using the default settings of RNA folding (version 2.3 energies). Because the maximum limit for mfold is 9000 bases, 2460 bases were removed between nucleotides 2278 and 4917 before folding the sequences. The removal of this sequence was based on an E–NS3 in-frame deletion that was observed in P5 of Torö-6A. To enhance the resolution of secondary structures in the 3′ and 5′NCRs of both viruses, the 5′ 706 nucleotides and the 3′ 701/733 nucleotides were folded. Torö-6A and Torö-38A variants with deletions in their 3′NCRs were individually folded using the same approach.

### Statistical analysis

The levels of the viral RNA in Vero B4 cells were analysed using 2-way ANOVA, and survival rates were analysed by log-rank analysis. The results are expressed as mean ± standard errors of the means (SEM).

## Additional Information

**How to cite this article**: Asghar, N. *et al*. The role of the poly(A) tract in the replication and virulence of tick-borne encephalitis virus. *Sci. Rep.* **6**, 39265; doi: 10.1038/srep39265 (2016).

**Publisher's note:** Springer Nature remains neutral with regard to jurisdictional claims in published maps and institutional affiliations.

## Supplementary Material

Supplementary Table and Data

## Figures and Tables

**Table 1:** Deletions within the 3′NCR of Torö-6A and Torö-38A from cell culture and mice.

	Position^a^	Size (nt)	Del (N)	Avg Cov	% Del
Torö-6A					
P0	10489	1	1554	2378	65
M1	10489	1	1659	1726	96
M2	10489	1	405	1786	23
M2	10495	1	1290	1827	71
M3	10489	1	1572	1685	93
M3	10495	1	66	1732	4
Torö-38A					
P0	10450–588	139	21	303	6.9
P0	10527–592	66	25	274	9.1
M1	10442–599	158	4	188	2.1
M1	10527–592	66	40	188	21.3
M2	10442–599	158	125	510	24.5
M3	10527–592	66	6	393	1.53

^a^The positions correspond to GenBank accession no. DQ401140.2. Abbreviations: P0, passage 0 (rescued virus); M, mouse; nt, nucleotide; Del (N), number of deletions; Avg Cov, average coverage; % Del, per cent deletions.

**Table 2:** Percentages of single nucleotide polymorphisms (>2%, except position 7967) within the open reading frame of Torö-6A and Torö-38A isolated from cell culture and mouse brains compared to GenBank accession no. DQ401140.2.

Position	Mutation	Region	A.A. change	Torö-6A							Torö-38A			
				**P0**	**P5-1**	**P5-2**	**P5-3**	**M1**	**M2**	**M3**	**P0**	**M1**	**M2**	**M3**
1504	G to A	E	Glu to Lys	18										
1674	C to A	E	Asn to Lys	30	100	100	100							
1847	T to C	E	Val to Ala								90	99	100	100
1930	G to A	E	Asp to Asn	19										
4104	G to A	NS2A	None	94	100	100	100	100	100	100	87	100	100	100
4104	G to T	NS2A	Lys to Asn	6							13			
4258	G to A	NS2B	Gly to Ser	4							11			
6278	C to T	NS3	Thr to Met								7	22	15	
6423	T to C	NS3	None	100	100	100	100	100	100	100	100	100	100	100
7462	G to T	NS4B	Val to Leu					97	100	72				
7967	G to A	NS5	Arg to Lys	22	44	42	1							
8282	G to C	NS5	Gly to Ala									2		2
8549	T to C	NS5	Met to Thr		2		6							
9480	G to A	NS5	None	74	100	100	100							

The percentage is based on read coverage ranging from 963 to 3322 reads per position. Abbreviations: A.A., amino acid; P, passage number; M, mouse.
