# Supplementary material for: The role of the poly(A) tract in the replication and virulence of tick-borne encephalitis virus
Source: Sci Rep. 2016 Dec 16;6:39265. doi: 10.1038/srep39265 (PMC5159820; doi:10.1038/srep39265)
Supplement: Supplementary Table and Data [file srep39265-s1.pdf]

The role of poly(A) tract in replication and virulence of tick-borne encephalitis virus

Naveed Asghar, Yi-Ping Lee, Emma Nilsson, Richard Lindqvist, Wessam Melik, Andrea Kröger, Anna K. Överby and Magnus Johansson

				**Torö-6A**							**Torö-38A**			
Position	Mutation	Region	A.A change	P0	P5-1	P5-2	P5-3	M1	M2	M3	P0	M1	M2	M3
36	A to G	5 NCR	N/A				2							
44	C to T	5 NCR	N/A										2	
90	A to G	5 NCR	N/A							2				
102	A to G	5 NCR	N/A				2							
114	A to G	5 NCR	N/A					25						
269	T to C	C	Leu to Pro					2						
366	C to T	C	None							6				
491	G to A	prM	Arg to Lys							2				
661	G to A	prM	Asp to Asn					3						
957	A to G	prM	None	15										
1026	T to C	E	None									4		
1039	T to C	E	None										2	
1040	T to C	E	Leu to Ser							5				
1068	C to T	E	None							9				
1118	A to T	E	Tyr to Phe				2							
1196	C to T	E	Pro to Leu									3		
1318	T to C	E	Cys to Arg									2		
1371	C to T	E	None		6									
1385	T to C	E	Val to Ala										2	

1487	C to T	E	Thr to Ile									6				
1504	G to A	E	Glu to Lys	18												
1554	G to A	E	None							2						
1586	C to T	E	Thr to Ile											6		
1674	C to A	E	Asn to Lys	30	100		100		100							
1686	A to G	E	None											5		
1847	T to C	E	Val to Ala										90	99	100	100
1930	G to A	E	Asp to Asn	19												
1997	T to C	E	Val to Ala										3			
2041	C to T	E	None					9								
2066	A to G	E	Glu to Gly											4		
2112	T to C	E	None					2								
2238	C to T	E	None											3		
2256	C to T	E	None							2						
2279	C to T	E	Ala to Val							3						
2532	G to T	NS1	Glu to Asp			3										
2888	A to G	NS1	Glu to Gly							2						
2966	T to C	NS1	Leu to Ser					2								
3045	C to T	NS1	None										3			
3107	T to C	NS1	Leu to Pro			2										
3452	G to A	NS1	Trp to *									5				
3545	G to A	NS2A	Gly to Glu					2								
3552	C to G	NS2A	None											7		
3559	G to A	NS2A	Val to Met			2										
3583	T to C	NS2A	Tyr to His			2										
3634	A to T	NS2A	Ile to Phe										10			
3817	C to T	NS2A	Leu to Phe								4					

3866	A to G	NS2A	Glu to Gly		2									
3983	T to C	NS2A	Leu to Ser			2								
3997	A to G	NS2A	Met to Val		2									
4104	G to A	NS2A	None	94	100	100	100	100	100	100	87	100	100	100
4104	G to T	NS2A	Lys to Asn	6							13			
4143	G to T	NS2A	None			7								
4215	T to C	NS2B	None			4								
4246	A to G	NS2B	Thr to Ala				29							
4258	G to A	NS2B	Gly to Ser	4							11			
4300	G to A	NS2B	Ala to Thr		16									
4389	T to C	NS2B	None						4					
4494	T to C	NS2B	None						2					
4557	G to A	NS2B	Met to Ile			3								
4780	C to T	NS3	None								2			
5016	C to T	NS3	None							4			4	
5028	C to A	NS3	None										2	
5160	C to T	NS3	None	19										
5238	C to A	NS3	None										3	
5418	C to T	NS3	None	11										
5436	G to A	NS3	None		6									
5511	T to G	NS3	None	23										
5688	C to T	NS3	None										2	
5997	G to A	NS3	None					10						
6070	A to G	NS3	Lys to Glu						2					
6159	G to A	NS3	None											6
6278	C to T	NS3	Thr to Met								7	22	15	
6315	T to C	NS3	None		4									

6333	T to C	NS3	None		4									
6423	T to C	NS3	None	100	100	100	100	100	100	100	100	100	100	100
6852	C to T	NS4A	None						76					
6894	T to C	NS4A	None										2	
7062	T to C	NS4B	None										2	
7462	G to T	NS4B	Val to Leu					97	100	72				
7493	C to T	NS4B	Thr to Ile		12									
7553	C to A	NS4B	Thr to Lys					3						
7671	T to C	NS5	None											2
7829	T to G	NS5	Val to Gly									23		
7938	G to A	NS5	None			7								
7967	G to A	NS5	Arg to Lys	22	44	42	1							
8282	G to C	NS5	Gly to Ala									2		2
8298	T to C	NS5	None									2		
8326	T to C	NS5	Tyr to His					5						
8339	C to T	NS5	Ala to Val							4				
8442	G to A	NS5	None					2						
8478	C to T	NS5	None					4						
8549	T to C	NS5	Met to Thr		2		6							
8568	C to T	NS5	None						9					
8764	C to T	NS5	Pro to Ser									2		
8997	C to T	NS5	None		12									
9081	T to C	NS5	None											2
9128	A to G	NS5	Glu to Gly									4		
9198	A to G	NS5	Ile to Met							3				
9447	C to T	NS5	None							3				
9450	C to T	NS5	None						9					

9480	G to A	NS5	None	74	100	100	100							
9528	G to A	NS5	None						8					
9531	C to T	NS5	None				3							
9555	C to T	NS5	None											2
9684	C to T	NS5	None					3						
9709	C to T	NS5	None						9					
10179	G to A	NS5	None									2		
10192	G to A	NS5	Ala to Thr								7			
10264	G to A	NS5	Ala to Thr							2				
10418	A to G	3 NCR	N/A									6		
10449	A to C	3 NCR	N/A								4			
10466	T to C	3 NCR	N/A										6	
10471	C to T	3 NCR	N/A								2			
10484	T to C	3 NCR	N/A								2			
10485	A to T	3 NCR	N/A								3			
10488	C to A	3 NCR	N/A								6	2	3	
10521	A to G	3 NCR	N/A										21	
10522	A to G	3 NCR	N/A									100		
10524	A to G	3 NCR	N/A								60		59	29
10526	A to G	3 NCR	N/A								7		3	
10495	G to T	3 NCR	N/A								6			
10502	T to C	3 NCR	N/A											2
10505	A to C	3 NCR	N/A								3			
10506	G to T	3 NCR	N/A								3			
10546	C to T	3 NCR	N/A					25						
10553	T to G	3 NCR	N/A								2			
10554	G to A	3 NCR	N/A								2			

10554	G to T	3 NCR	N/A					2			
10556	C to A	3 NCR	N/A						12		
10557	A to G	3 NCR	N/A						6		
10559	A to G	3 NCR	N/A		2						
10567	G to T	3 NCR	N/A					18			
10599	T to C	3 NCR	N/A				3				
10643	C to T	3 NCR	N/A						4		
10678	G to T	3 NCR	N/A	21							
10694	G to A	3 NCR	N/A						2		
10712	C to T	3 NCR	N/A								3
10759	A to G	3 NCR	N/A							2	7
10808	G to A	3 NCR	N/A	14							
11005	A to G	3 NCR	N/A						2		
11016	T to C	3 NCR	N/A	13							

Supplementary Table S1. Percent[a] single nucleotide polymorphism (>2% except position 7967) within open reading frame of Torö-6A and Torö-38A isolated from cell culture and mouse brains compared to GenBank accession no. DQ401140.2.

[a]The percent is based on average depth of 1548 and 1394 reads per position obtained for Torö-6A and Torö-38A, respectively. The positions in red correspond to Torö-38A. Abbreviations: A.A, amino acid; M, mice; N/A, not applicable; P, passage number.

Supplementary data 1

Chromatogram showing sequencing in forward direction of a Torö-38A clone in pcDNA3.1 vector having 57A long poly(A) tract. The nucleotide positions 648-704 bp correspond to the poly(A) tract.

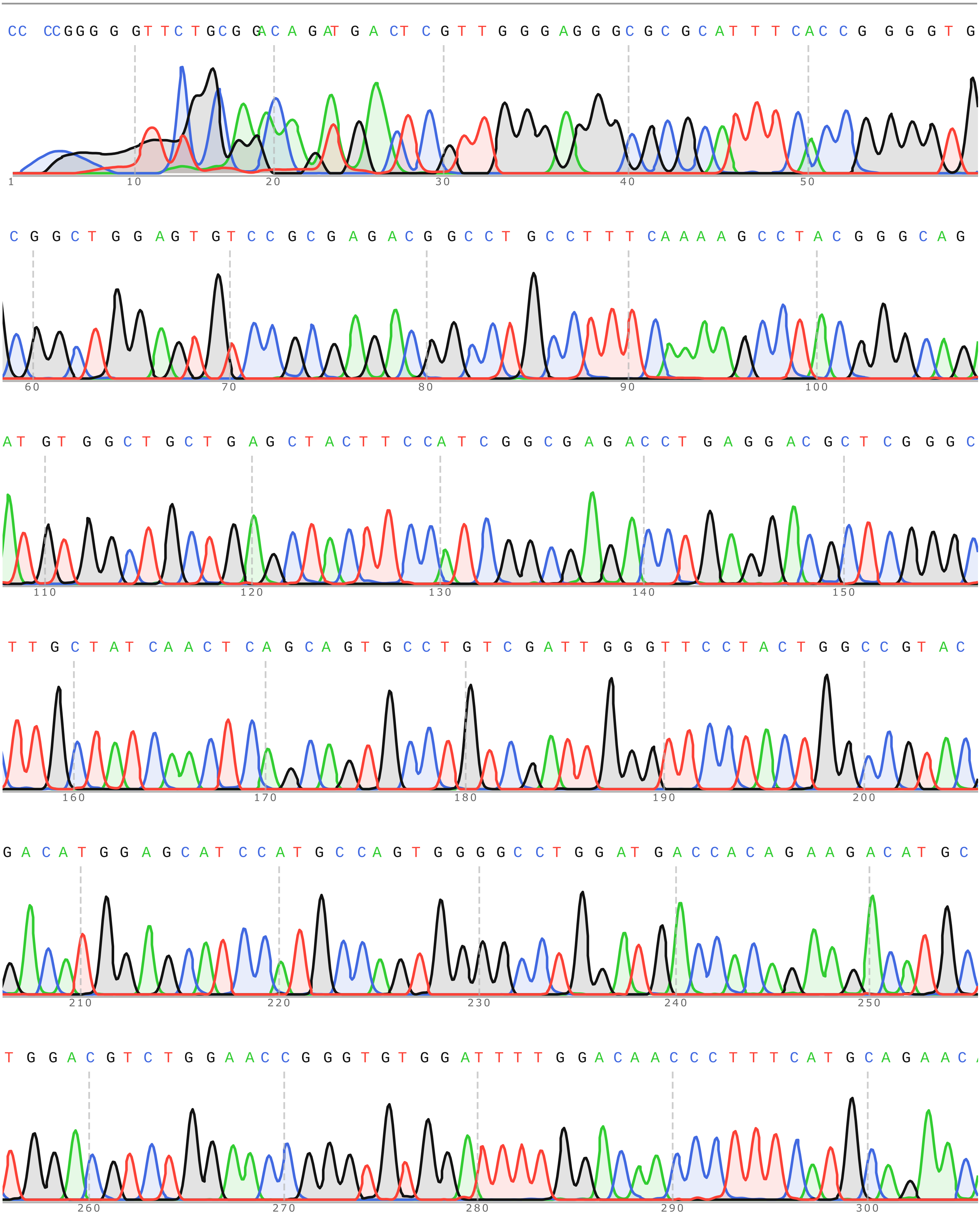
CC CCGGG G GTTCTGCG GACA GAT G A CT C G T T G G G A G G G C G C G C A T T T C A C C G G G G T G
C G G C T G G A G T G T C C G C G A G A C G G C C T G C C T T T C A A A A G C C T A C G G G C A G
AT GT G G C T G C T G A G C T A C T T C C A T C G G C G A G A C C T G A G G A C G C T C G G G C
T T G C T A T C A A C T C A G C A G T G C C T G T C G A T T G G G T T C C T A C T G G C C G T A C
G A C A T G G A G C A T C C A T G C C A G T G G G G C C T G G A T G A C C A C A G A A G A C A T G C
T G G A C G T C T G G A A C C G G G T G T G G A T T T T G G A C A A C C C T T T C A T G C A G A A C

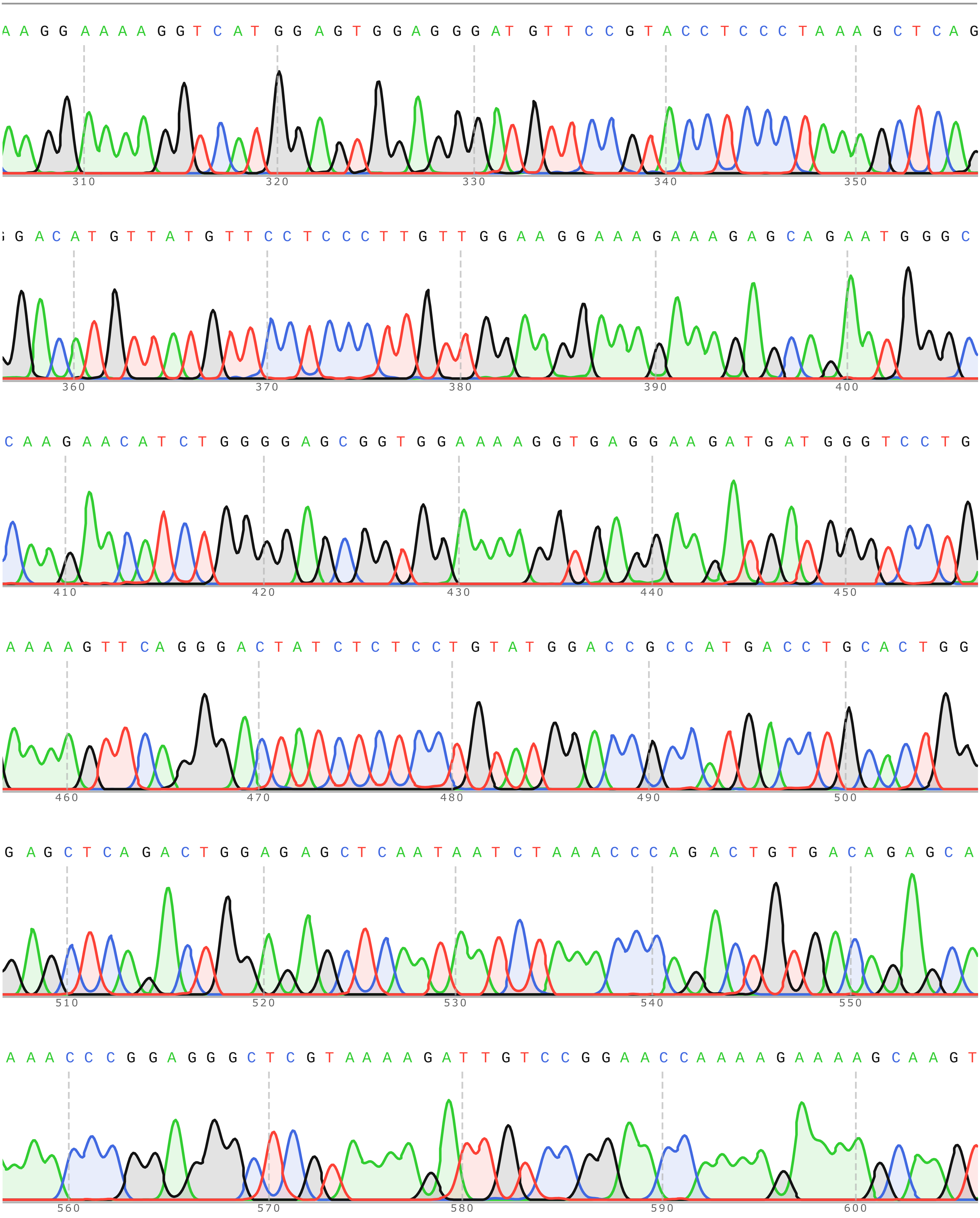
A A G G A A A A G G T C A T G G A G T G G A G G G A T G T T C C G T A C C T C C C T A A A G C T C A G
310 320 330 340 350
G A C A T G T T A T G T T C C T C C C T T G T T G G A A G G A A A G A A A G A G C A G A A T G G G C
360 370 380 390 400
C A A G A A C A T C T G G G G A G C G G T G G A A A A G G T G A G G A A G A T G A T G G G T C C T G
410 420 430 440 450
A A A A G T T C A G G G A C T A T C T C T C C T G T A T G G A C C G C C A T G A C C T G C A C T G G
460 470 480 490 500
G A G C T C A G A C T G G A G A G C T C A A T A A T C T A A A C C C A G A C T G T G A C A G A G C A
510 520 530 540 550
A A A C C C G G A G G G C T C G T A A A A G A T T G T C C G G A A C C A A A A G A A A A G C A A G T
560 570 580 590 600

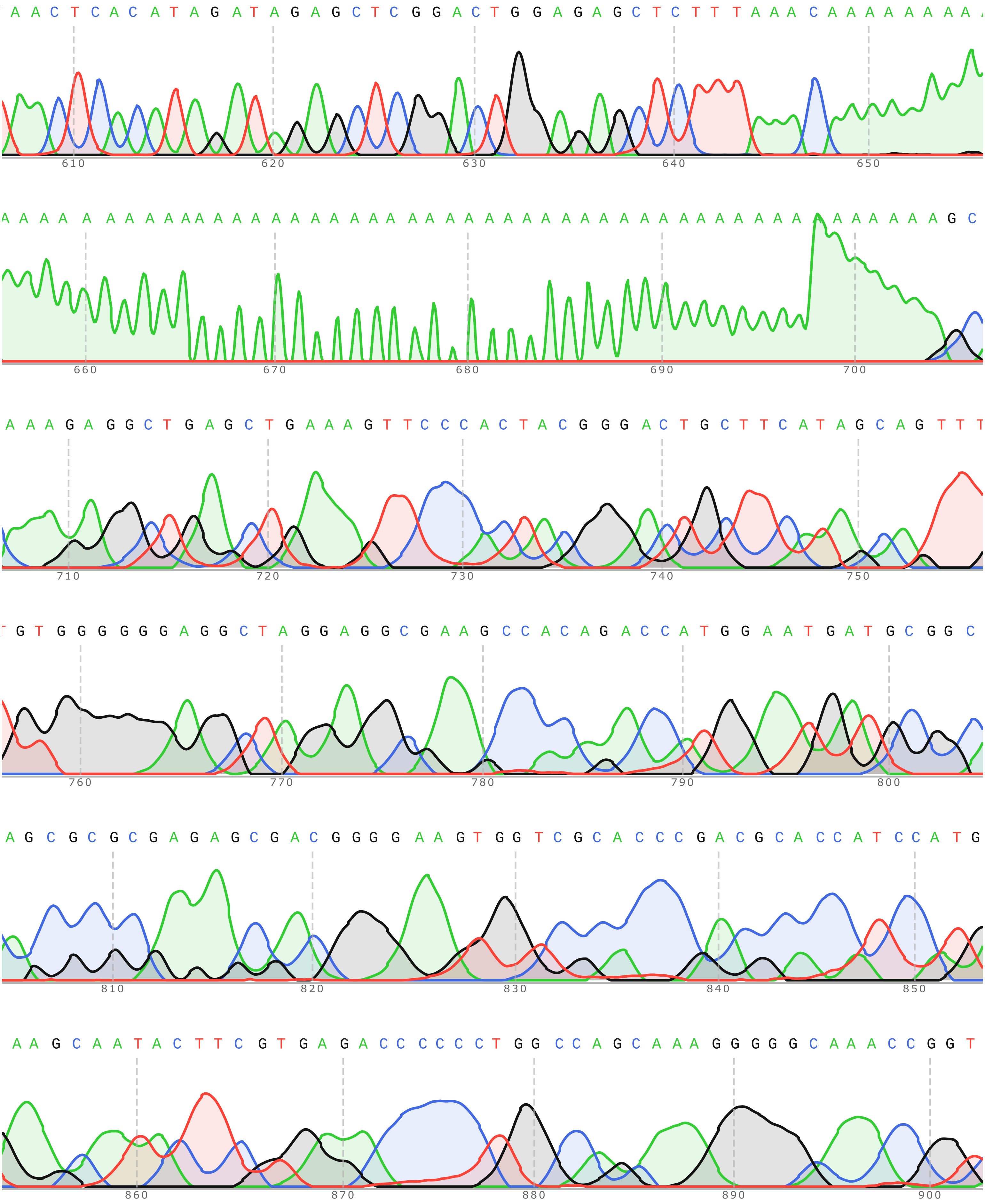
AACTCACATAGATAGAGCTCGGACTGGAGAGCTCTTTAAACAAAAAAAA
610 620 630 640 650
AAAAAAAAAAAAAAAAAAAAAAAAAAAAAAAAAAAAAAAAAAAAAAAAAGC
660 670 680 690 700
AAAGAGGCTGAGCTGAAAGTTCCCACTACGGGACTGCTTCATAGCAGTTT
710 720 730 740 750
TGTGGGGGGAGGCTAGGAGGCGAAGCCACAGACCATGGAATGATGCGGC
760 770 780 790 800
AGCGCGCGAGAGCGACGGGGAAGTGGTCGCACCCGACGCACCATCCATG
810 820 830 840 850
AAGCAATACTTCGTGAGACCCCCCTGGCCAGCAAAGGGGGCAAACCGGT
860 870 880 890 900

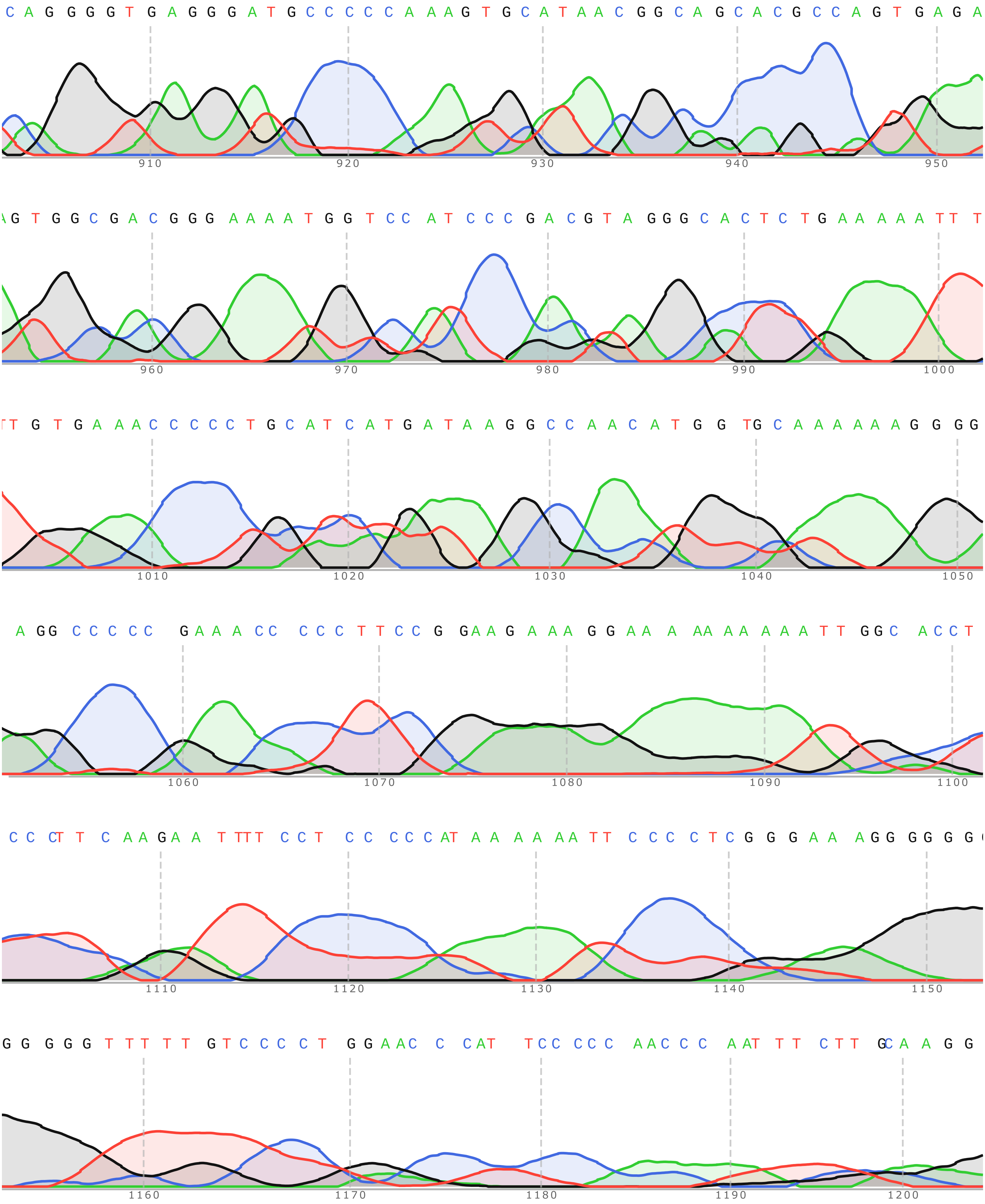
C A G G G G T G A G G G A T G C C C C C A A A G T G C A T A A C G G C A G C A C G C C A G T G A G A
910 920 930 940 950
A G T G G C G A C G G G A A A A T G G T C C A T C C C G A C G T A G G G C A C T C T G A A A A A T T T
960 970 980 990 1000
T T G T G A A A C C C C C T G C A T C A T G A T A A G G C C A A C A T G G T G C A A A A A A G G G G
1010 1020 1030 1040 1050
A G G C C C C C G A A A C C C C C T T C C G G A A G A A A G G A A A A A A A A A A T T G G C A C C T
1060 1070 1080 1090 1100
C C C T T C A A G A A T T T T C C T C C C C C A T A A A A A A T T C C C C T C G G G A A A G G G G G G
1110 1120 1130 1140 1150
G G G G G T T T T T G T C C C C T G G A A C C C A T T C C C C C A A C C C A A T T T C T T G C A A G G
1160 1170 1180 1190 1200

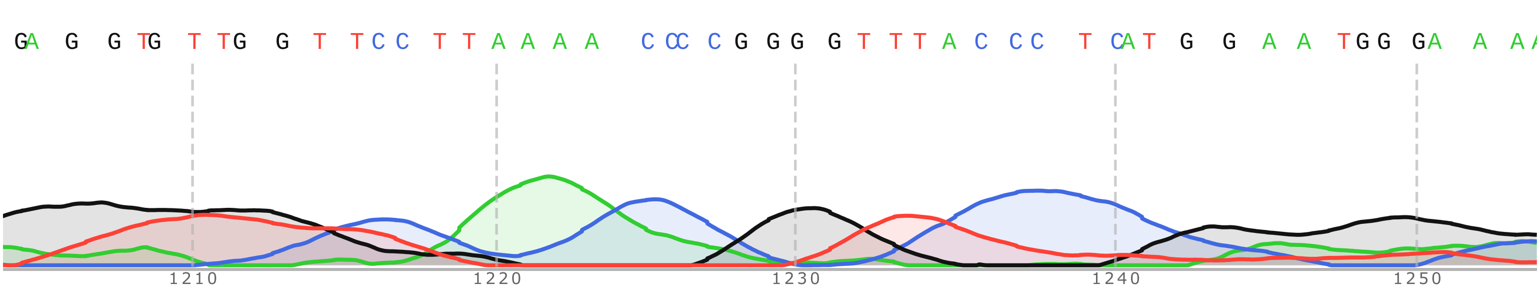
G A G G T G T T G G T T C C T T A A A A C C C C G G G G T T T A C C C T C A T G G A A T G G G A A A A
1210
1220
1230
1240
1250

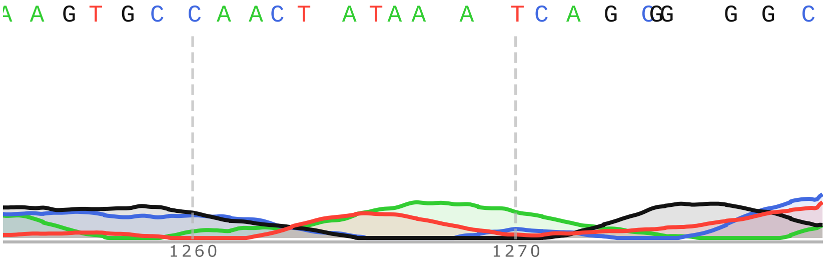
A A G T G C C A A C T A T A A A T C A G C G G G G C
1260
1270

Supplementary data 2

Chromatogram showing sequencing in reverse direction of a Torö-38A clone in pcDNA3.1 vector having 57A long poly(A) tract. The nucleotide positions 490-546 bp correspond to the poly(A) tract.

Supplementary File 2.scf (1080 bases)

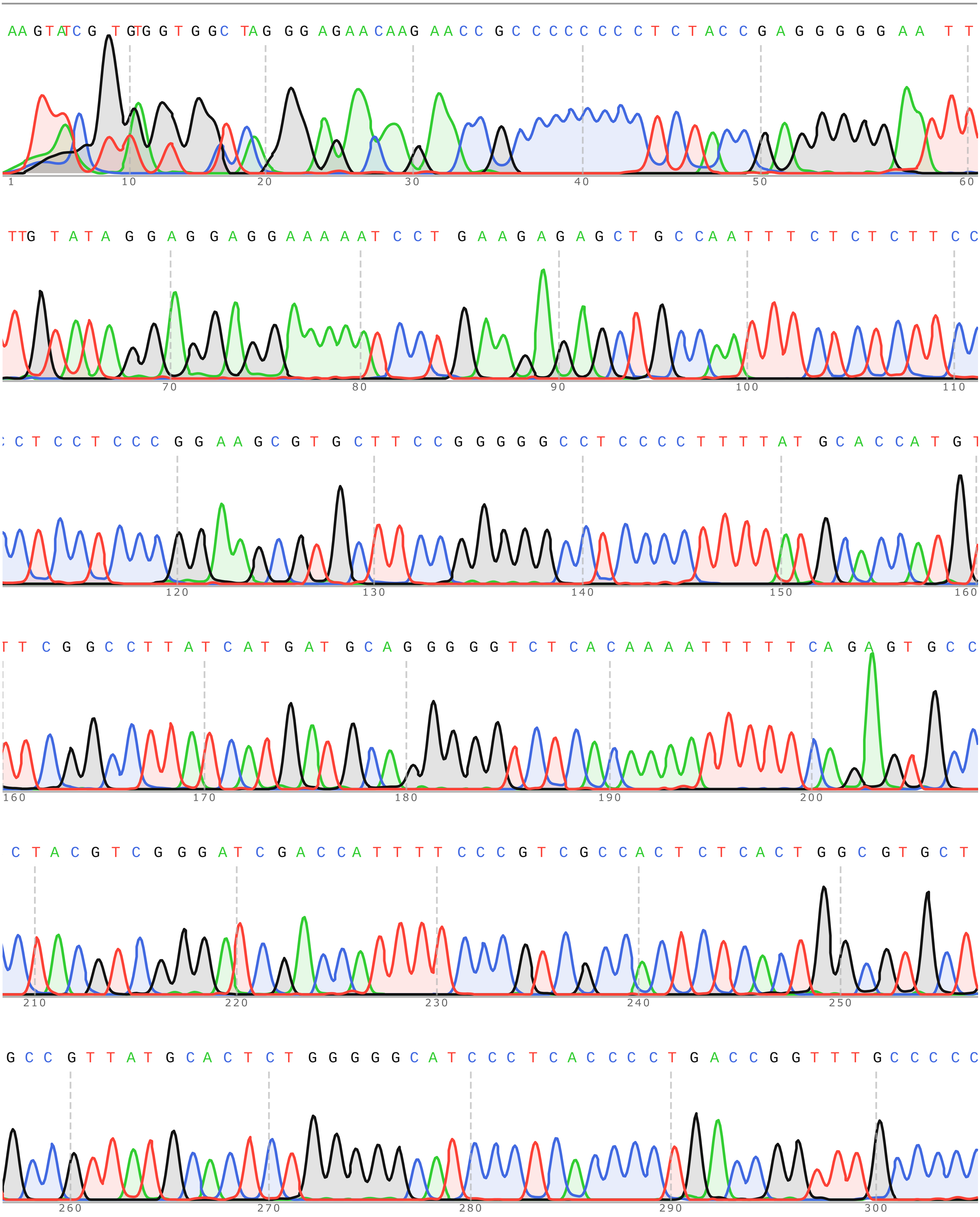

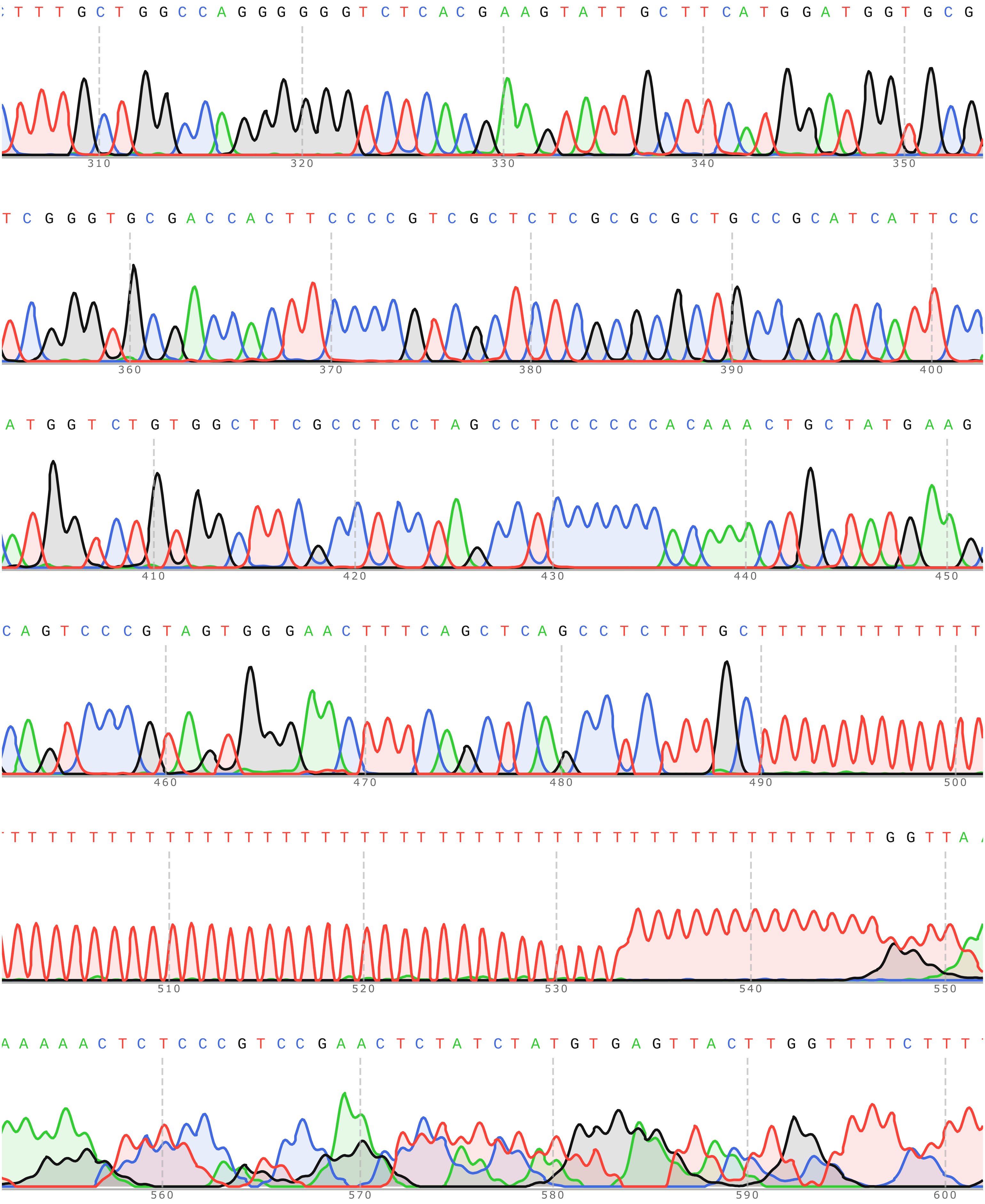
T T T G C T G G C C A G G G G G G T C T C A C G A A G T A T T G C T T C A T G G A T G G T G C G
310 320 330 340 350
T C G G G T G C G A C C A C T T C C C C G T C G C T C T C G C G C G C T G C C G C A T C A T T C C
360 370 380 390 400
A T G G T C T G T G G C T T C G C C T C C T A G C C T C C C C C A C A A A C T G C T A T G A A G
410 420 430 440 450
C A G T C C C G T A G T G G G A A C T T T C A G C T C A G C C T C T T T G C T T T T T T T T T T T T
460 470 480 490 500
T T T T T T T T T T T T T T T T T T T T T T T T T T T T T T T T T T T T T T T T T T T T T T T G G T T A
510 520 530 540 550
A A A A A C T C T C C C G T C C G A A C T C T A T C T A T G T G A G T T A C T T G G T T T T C T T T
560 570 580 590 600

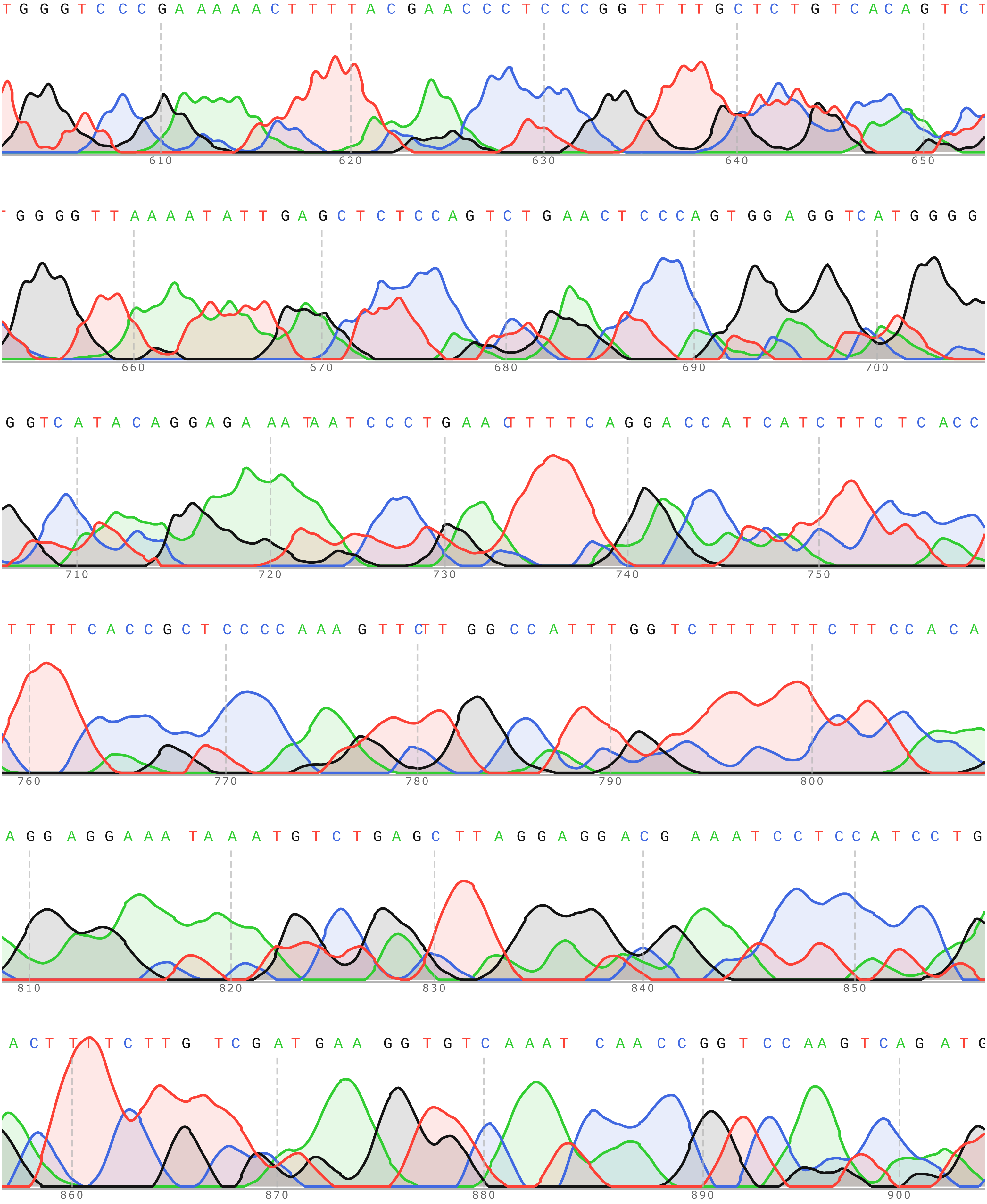
TGGGTCCCGAAAAACTTTTACGAACCCTCCCGGTTTTGCTCTGTCACAGTCT
610 620 630 640 650
TGGGGTTAAAATATTGAGCTCTCCAGTCTGAACTCCCAGTGGAGGTCATGGGG
660 670 680 690 700
GGTCATACAGGAGAAATAATCCCTGAACTTTTCAGGACCATCATCTTCTCACC
710 720 730 740 750
TTTTCACCGCTCCCCAAAGTTCTTGGCCATTTGGTCTTTTTTCTTCCACA
760 770 780 790 800
AGGAGGAAATAAATGTCTGAGCTTAGGAGGACGAAATCCTCCATCCTG
810 820 830 840 850
ACTTTTCTTGTCGATGAAGGTGTCAAATCAACCGGTCCAAGTCAGATG
860 870 880 890 900

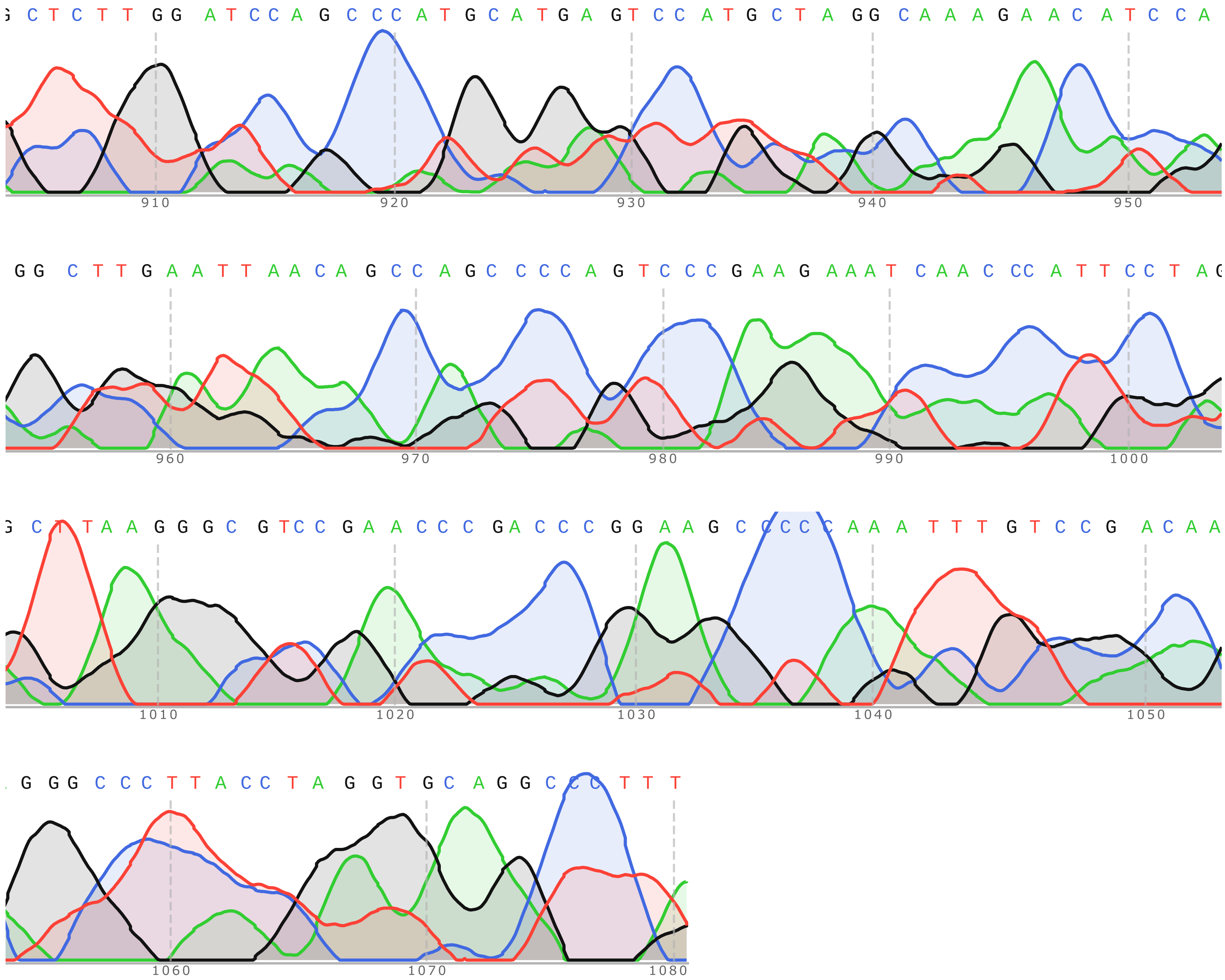
910
920
930
940
950
960
970
980
990
1000
1010
1020
1030
1040
1050
1060
1070
1080

Supplementary data 3

Chromatogram showing sequencing in forward direction of a Torö-38A clone in pcDNA3.1 vector having 49A long poly(A) tract. The nucleotide positions 646-794 bp correspond to the poly(A) tract.

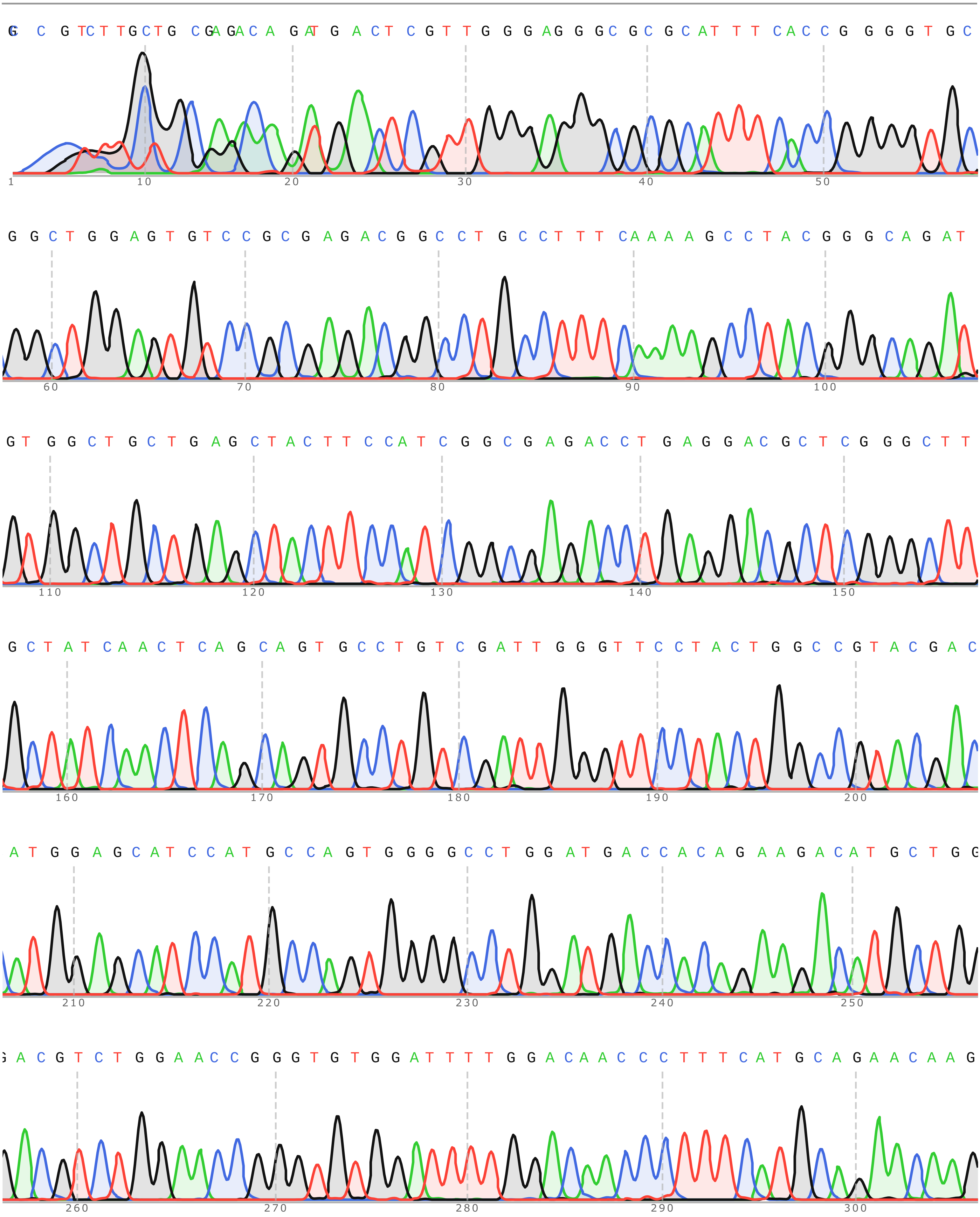
GCGTCTTGCTGCGAGACAGATGACTCGTTGGGAGGGCGCGCATTTCACCGGGGTGC
1 10 20 30 40 50
GGCTGGAGTGTCCGCGAGACGGCCTGCCTTTCAAAAGCCTACGGGCAGAT
60 70 80 90 100
GTGGCTGCTGAGCTACTTCCATCGGCGAGACCTGAGGACGCTCGGGCTT
110 120 130 140 150
GCTATCAACTCAGCAGTGCCTGTCGATTGGGTTCCTACTGGCCGTACGAC
160 170 180 190 200
ATGGAGCATCCATGCCAGTGGGGCCTGGATGACCACAGAAGACATGCTGG
210 220 230 240 250
ACGTCTGGAACCGGGTGTGGATTTTGGACAACCCTTTCATGCAGAACAAG
260 270 280 290 300

G A A A A G G T C A T G G A G T G G A G G G A T G T T C C G T A C C T C C C T A A A G C T C A G G A

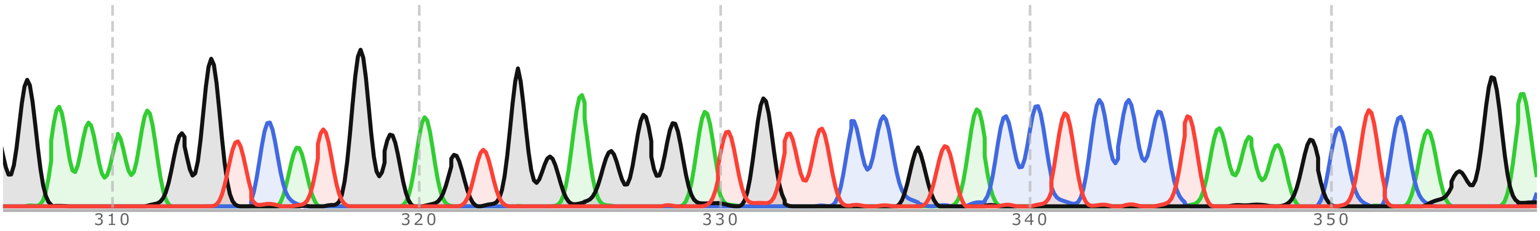

C A T G T T A T G T T C C T C C C T T G T T G G A A G G A A A G A A A G A G C A G A A T G G G C C A

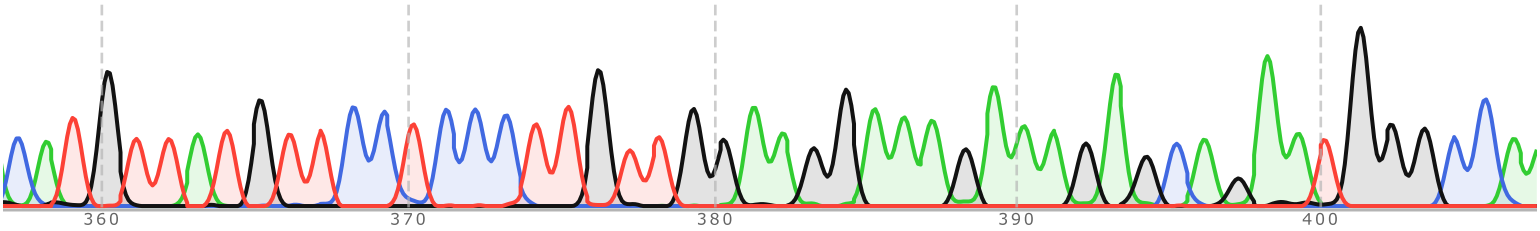

G A A C A T C T G G G G A G C G G T G G A A A A G G T G A G G A A G A T G A T G G G T C C T G A A

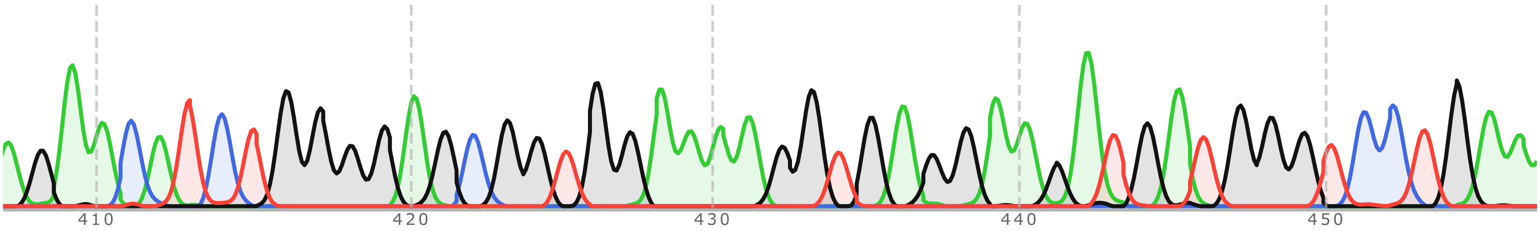

A A G T T C A G G G A C T A T C T C T C C T G T A T G G A C C G C C A T G A C C T G C A C T G G G A

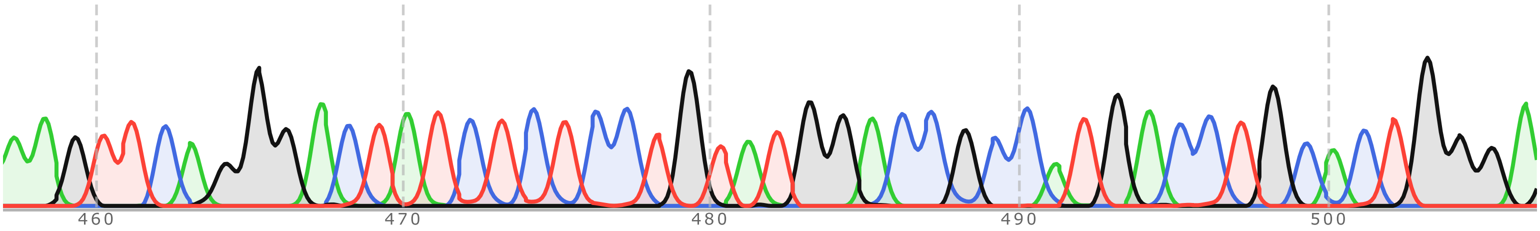

G C T C A G A C T G G A G A G C T C A A T A A T C T A A A C C C A G A C T G T G A C A G A G C A A A

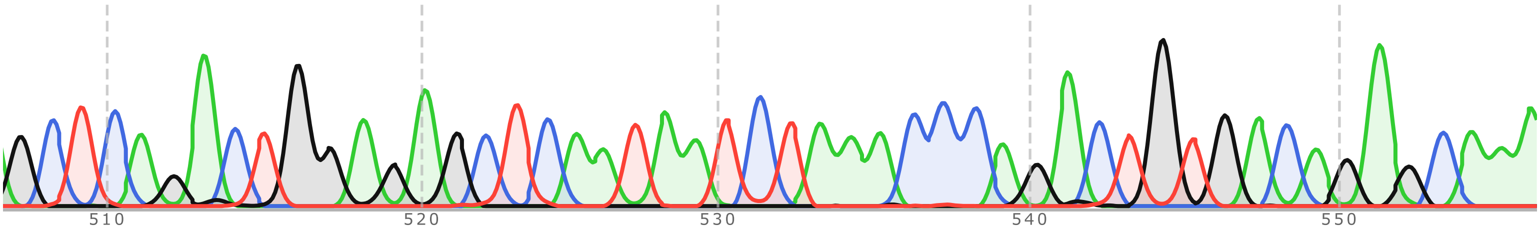

A C C C G G A G G G C T C G T A A A A G A T T G T C C G G A A C C A A A G G A A A A G C A A G T A

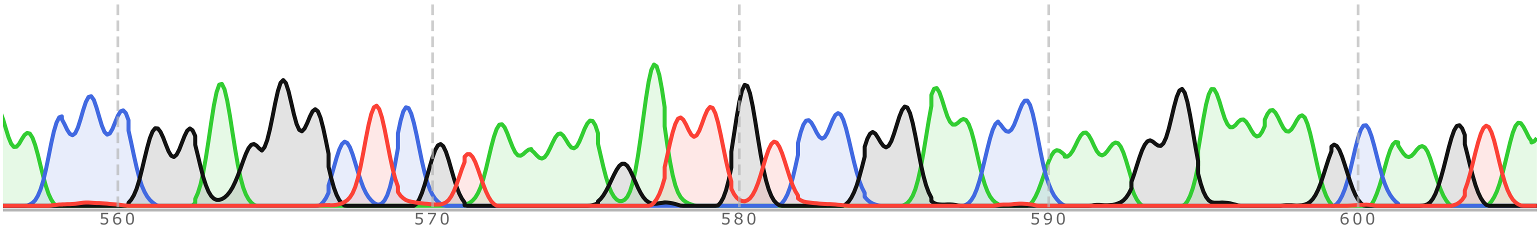

A C T C A C A T A G A T A G A G C T C G G A C T G G A G A G C T C T T T A A A C A A A A A A A A A

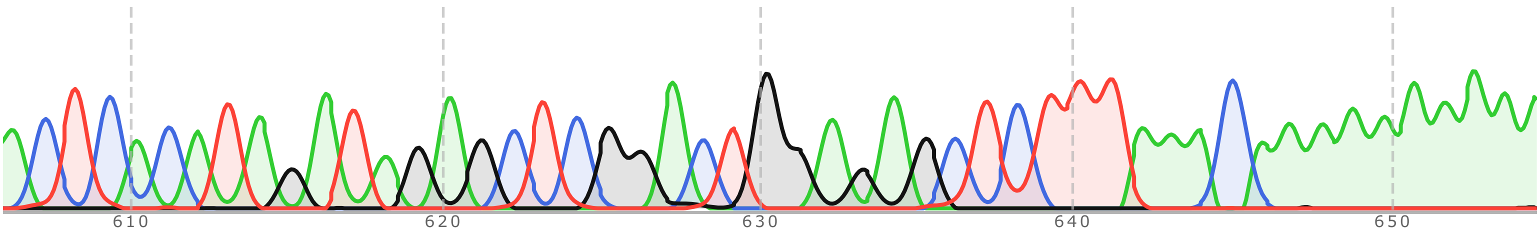

A A A A A A A A A A A A A A A A A A A A A A A A A A A A A A A A A A A A A A A A G C C A G A A T T G

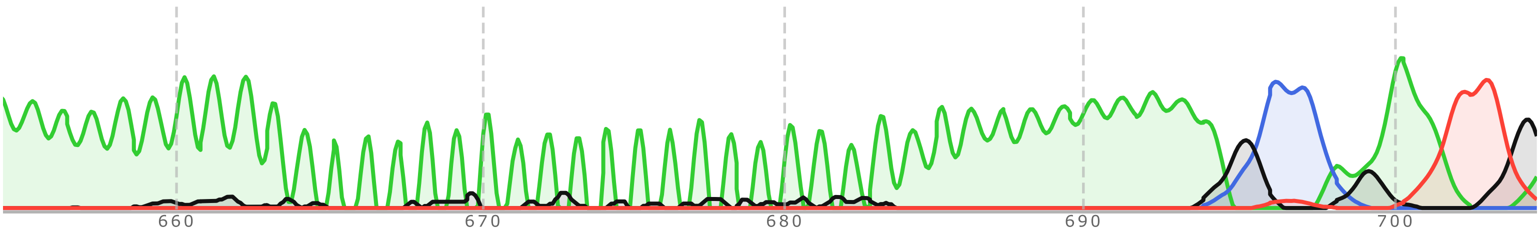

A G C T G A A C C T G G A G A G C T C A T T A A A C A T A G T C C A G A C A A A A C A A A A C A T

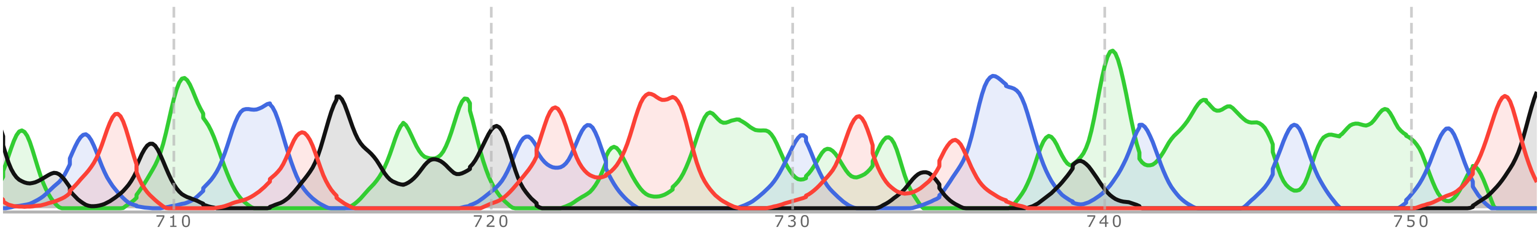

A C A A A G C A A A G A G G C T G A G C T G A A A G T T C C C A C T A C G G G A C T G C T T C A T

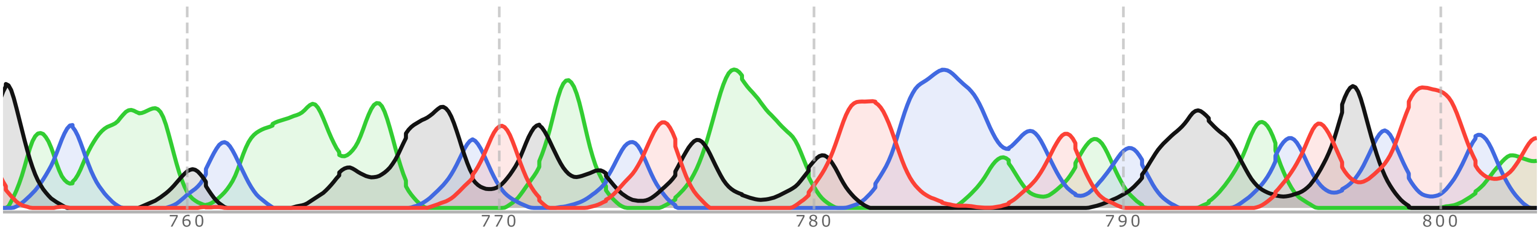

A G C A G T T T G T G G G G G A G G C T A G G A G G C G A A G C C A C A G A C C A T G G A A T

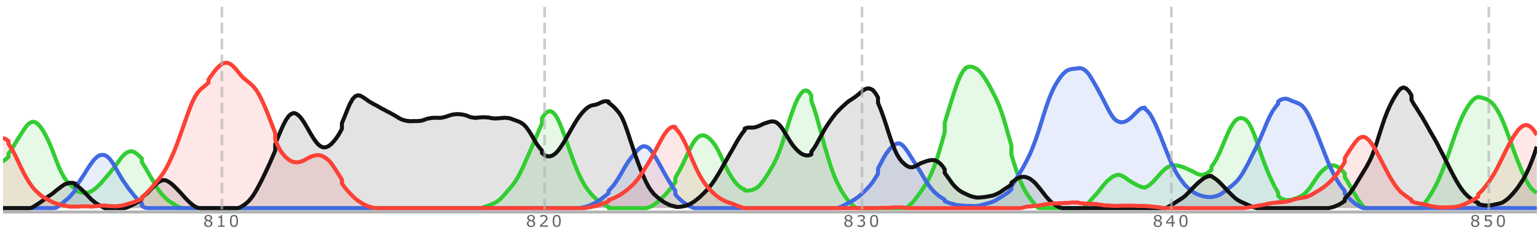

G A T G C G G C A G C G C G C G A G A G C G A C G G G G A A G T G G T C G C A C C C G A C G C A

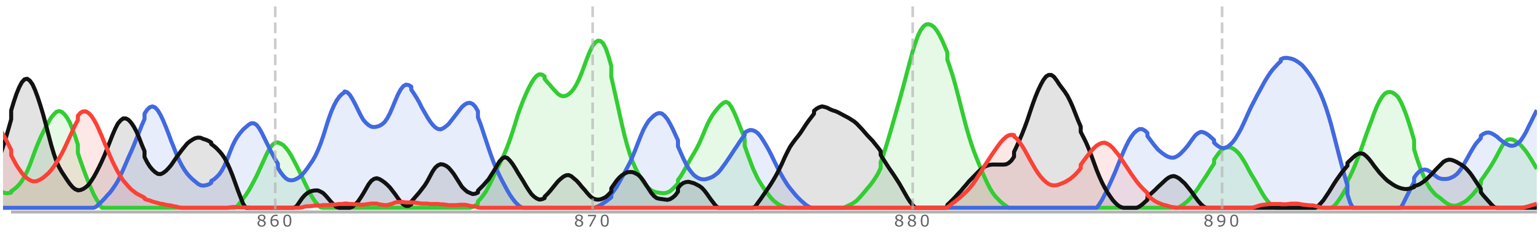

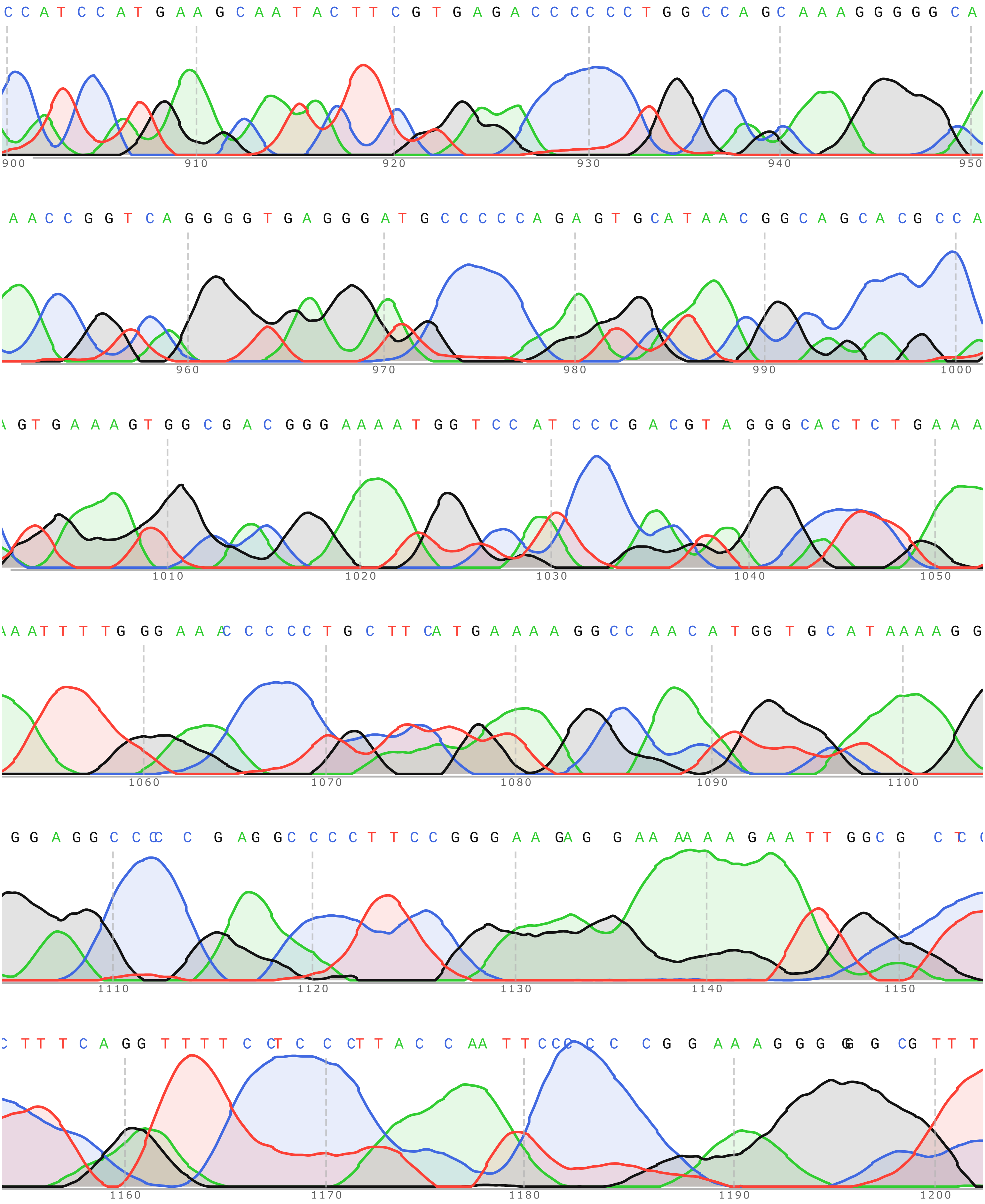
C C A T C C A T G A A G C A A T A C T T C G T G A G A C C C C C C T G G C C A G C A A A G G G G G C A
900 910 920 930 940 950
A A C C G G T C A G G G G T G A G G G A T G C C C C C A G A G T G C A T A A C G G C A G C A C G C C A
960 970 980 990 1000
A G T G A A A G T G G C G A C G G G A A A A T G G T C C A T C C C G A C G T A G G G C A C T C T G A A A
1010 1020 1030 1040 1050
A A A T T T T G G G A A A C C C C C T G C T T C A T G A A A A G G C C A A C A T G G T G C A T A A A A G G
1060 1070 1080 1090 1100
1110 1120 1130 1140 1150
1160 1170 1180 1190 1200

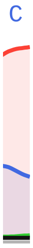
C

Supplementary data 4

Chromatogram showing sequencing in reverse direction of a Torö-38A clone in pcDNA3.1 vector having 49A long poly(A) tract. The nucleotide positions 554-603 bp correspond to the poly(A) tract.

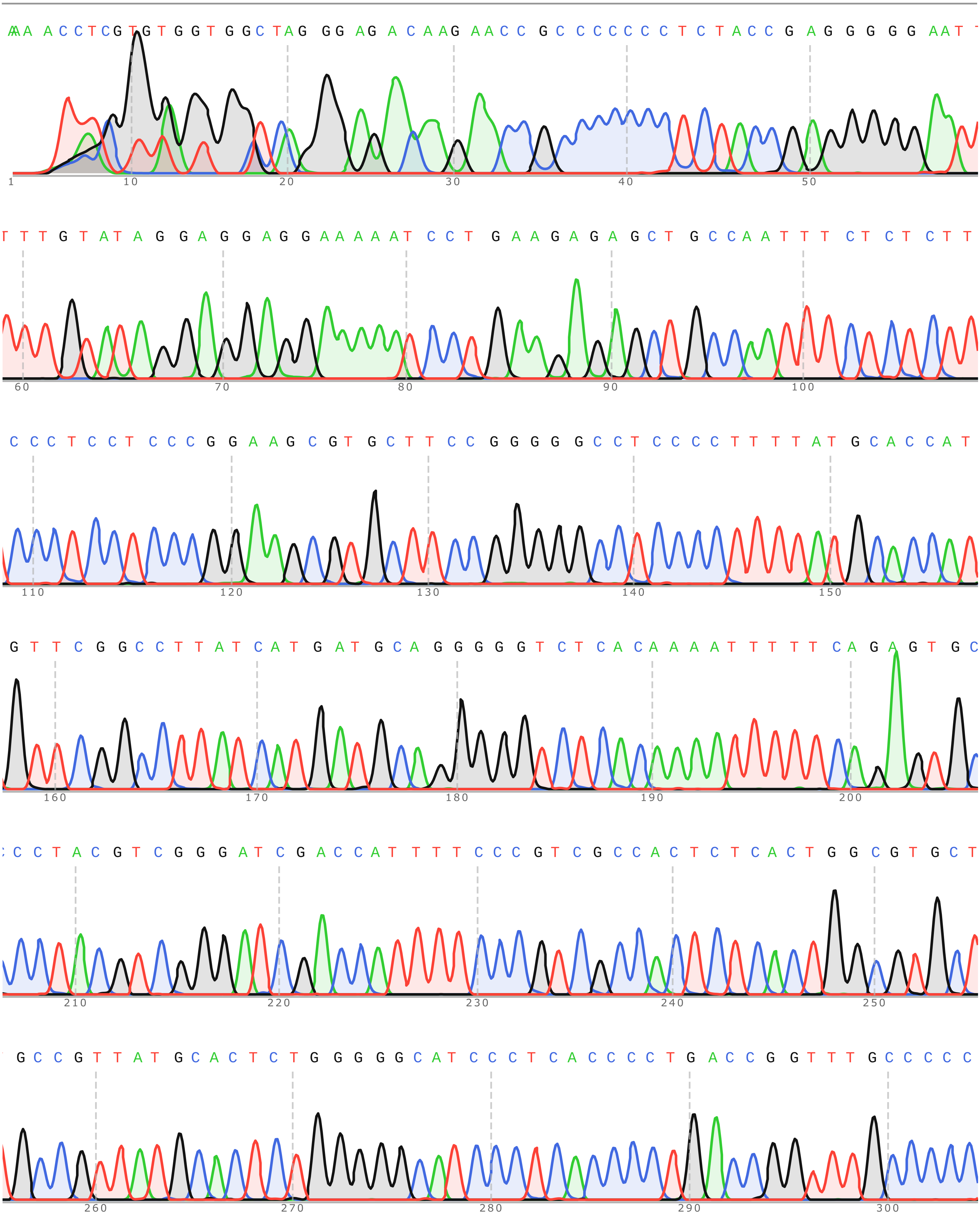
AAACCTCGTGTGGTGGCTAGGGAGACAAGAACCGCCCCCCCTCTACCGAGGGGGAATT
1 10 20 30 40 50
TTTGTATAGGAGGAGGAAAAATCCTGAAGAGAGCTGCCAATTTCTCTCTT
60 70 80 90 100
CCCTCCTCCCGGAAGCGTGCTTCCGGGGGCCTCCCCTTTTATGCACCAT
110 120 130 140 150
GTTCGGCCTTATCATGATGCAGGGGGTCTCACAAAATTTTTCAGAGTGC
160 170 180 190 200
CCTACGTCGGGATCGACCATTTTCCCGTCGCCACTCTCACTGGCGTGCT
210 220 230 240 250
GCCGTTATGCACTCTGGGGGCATCCCTCACCCCTGACCGGTTTGCCCCC
260 270 280 290 300

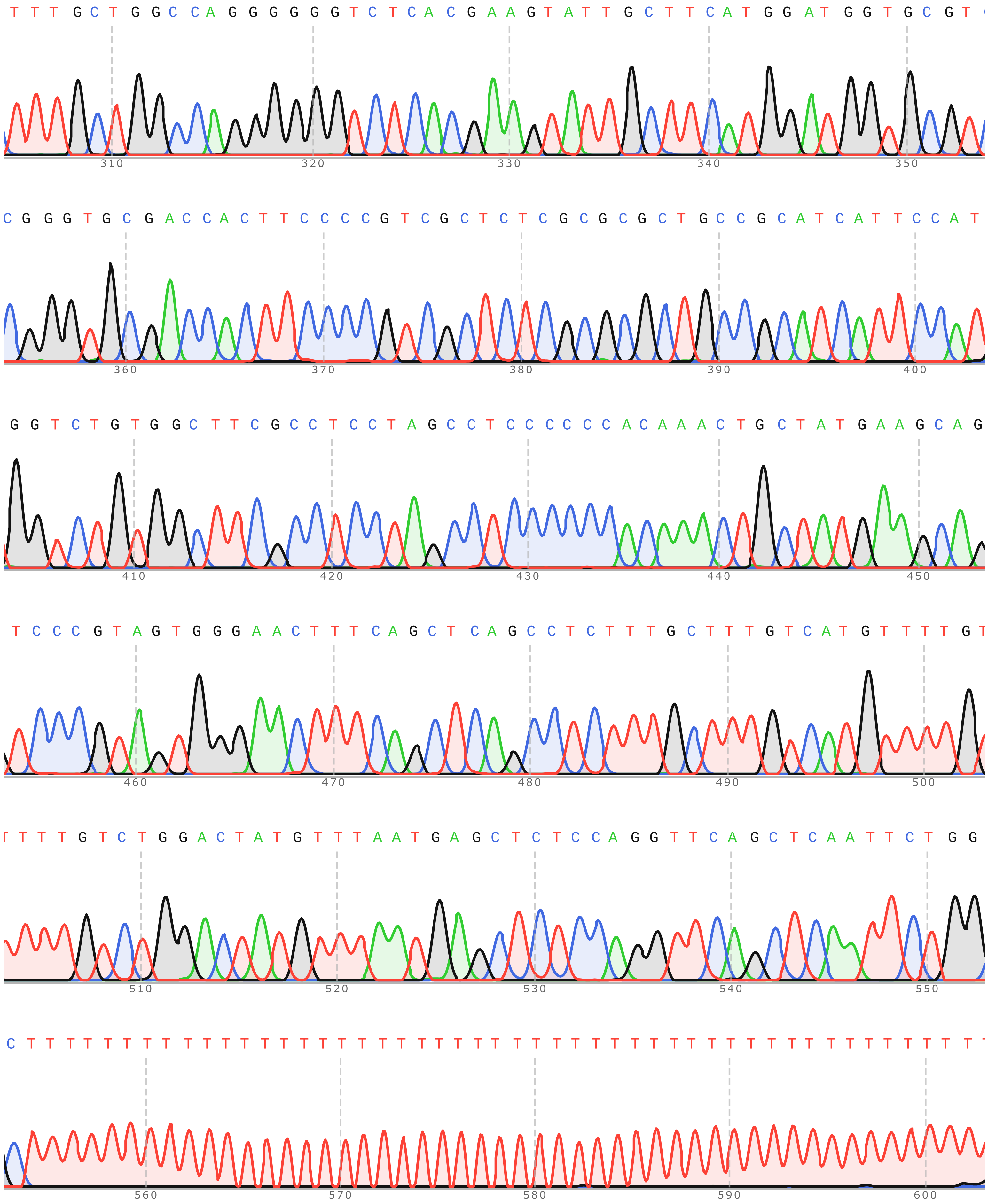
T T T G C T G G C C A G G G G G G T C T C A C G A A G T A T T G C T T C A T G G A T G G T G C G T
310 320 330 340 350
C G G G T G C G A C C A C T T C C C C G T C G C T C T C G C G C G C T G C C G C A T C A T T C C A T
360 370 380 390 400
G G T C T G T G G C T T C G C C T C C T A G C C T C C C C C A C A A A C T G C T A T G A A G C A G
410 420 430 440 450
T C C C G T A G T G G G A A C T T T C A G C T C A G C C T C T T T G C T T T G T C A T G T T T T G T
460 470 480 490 500
T T T G T C T G G A C T A T G T T T A A T G A G C T C T C C A G G T T C A G C T C A A T T C T G G
510 520 530 540 550
C T T T T T T T T T T T T T T T T T T T T T T T T T T T T T T T T T T T T T T T T T T T T T T T T T
560 570 580 590 600

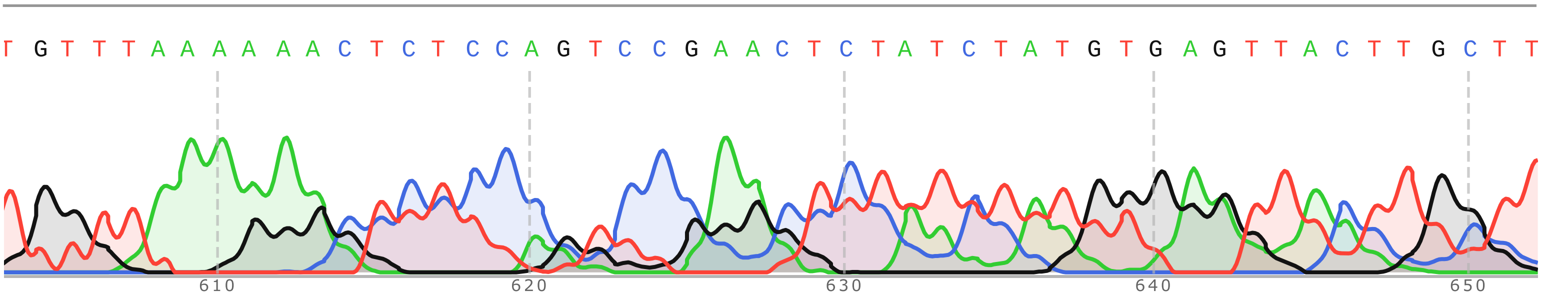
T G T T T A A A A A A C T C T C C A G T C C G A A C T C T A T C T A T G T G A G T T A C T T G C T T
610 620 630 640 650

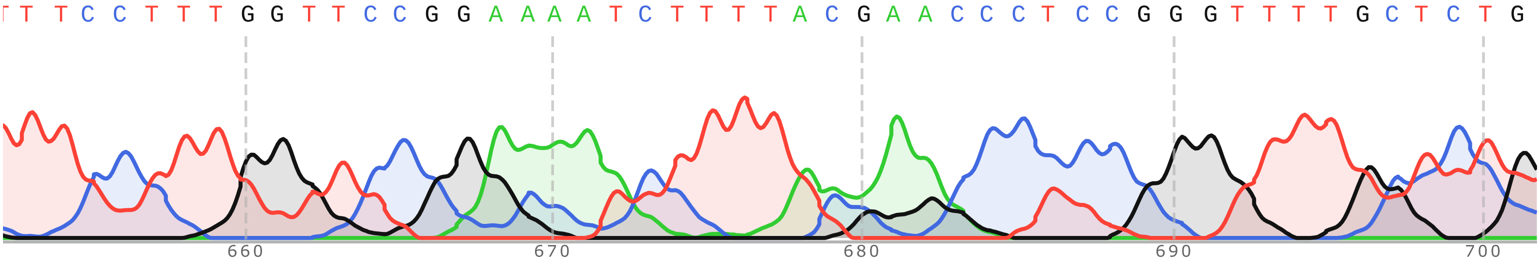
T T T C C T T T G G T T C C G G A A A A T C T T T T A C G A A C C C T C C G G G T T T T G C T C T G
660 670 680 690 700

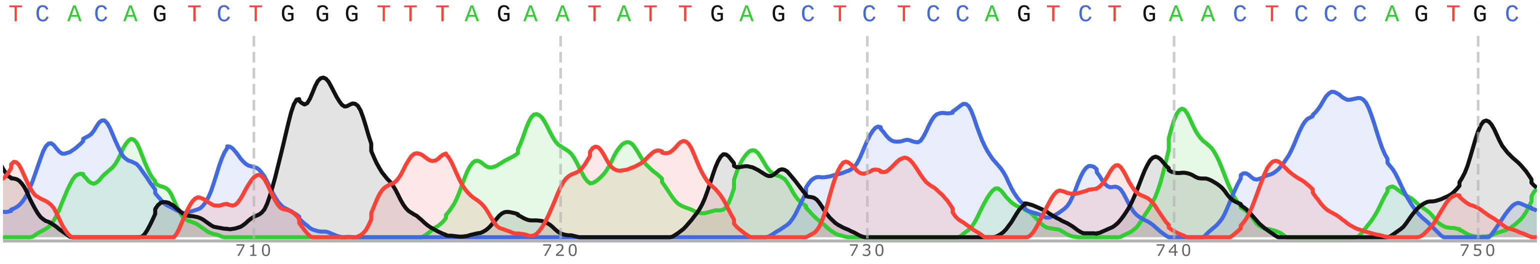
T C A C A G T C T G G G T T T A G A A T A T T G A G C T C T C C A G T C T G A A C T C C C A G T G C
710 720 730 740 750

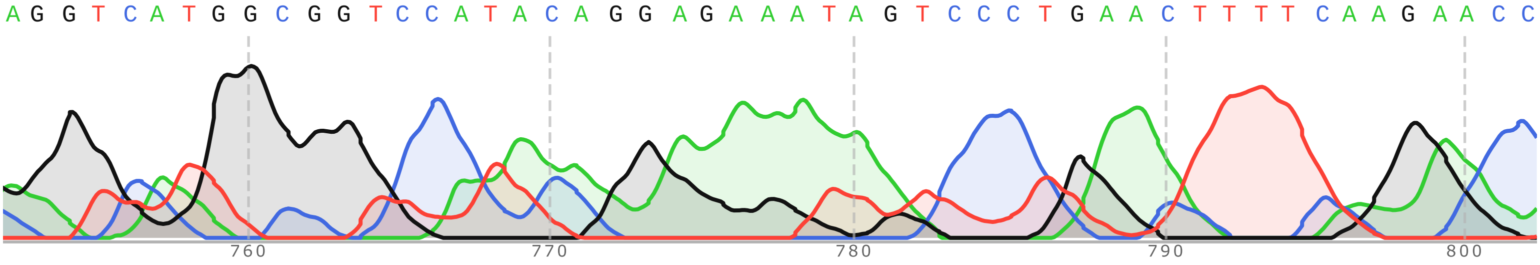
A G G T C A T G G C G G T C C A T A C A G G A G A A A T A G T C C C T G A A C T T T T C A A G A A C C
760 770 780 790 800

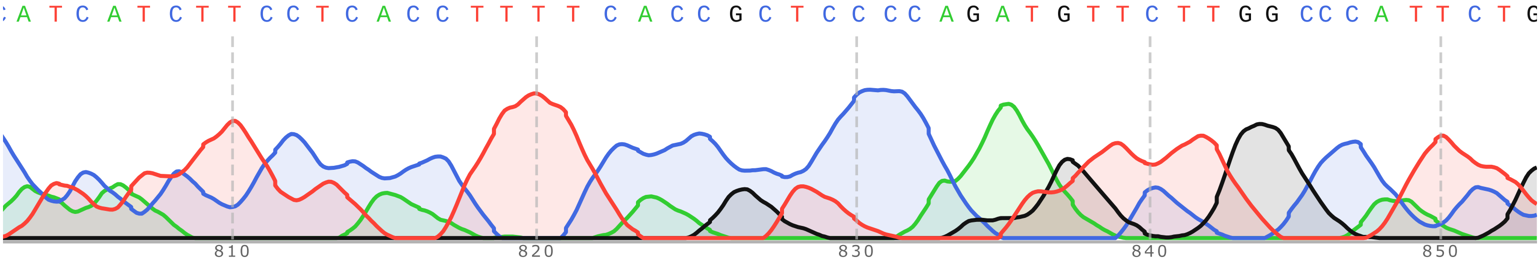
C A T C A T C T T C C T C A C C T T T T C A C C G C T C C C C A G A T G T T C T T G G C C C A T T C T G
810 820 830 840 850

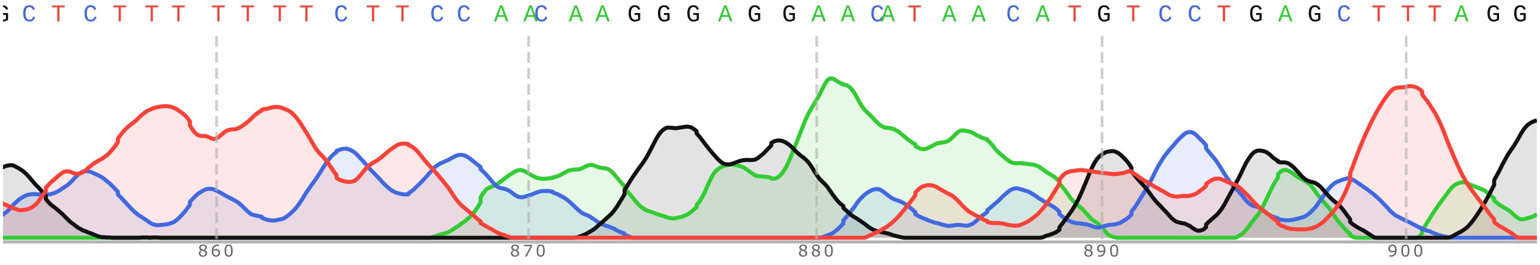
G C T C T T T T T T T C T T C C A A C A A G G G A G G A A C A T A A C A T G T C C T G A G C T T T A G G
860 870 880 890 900

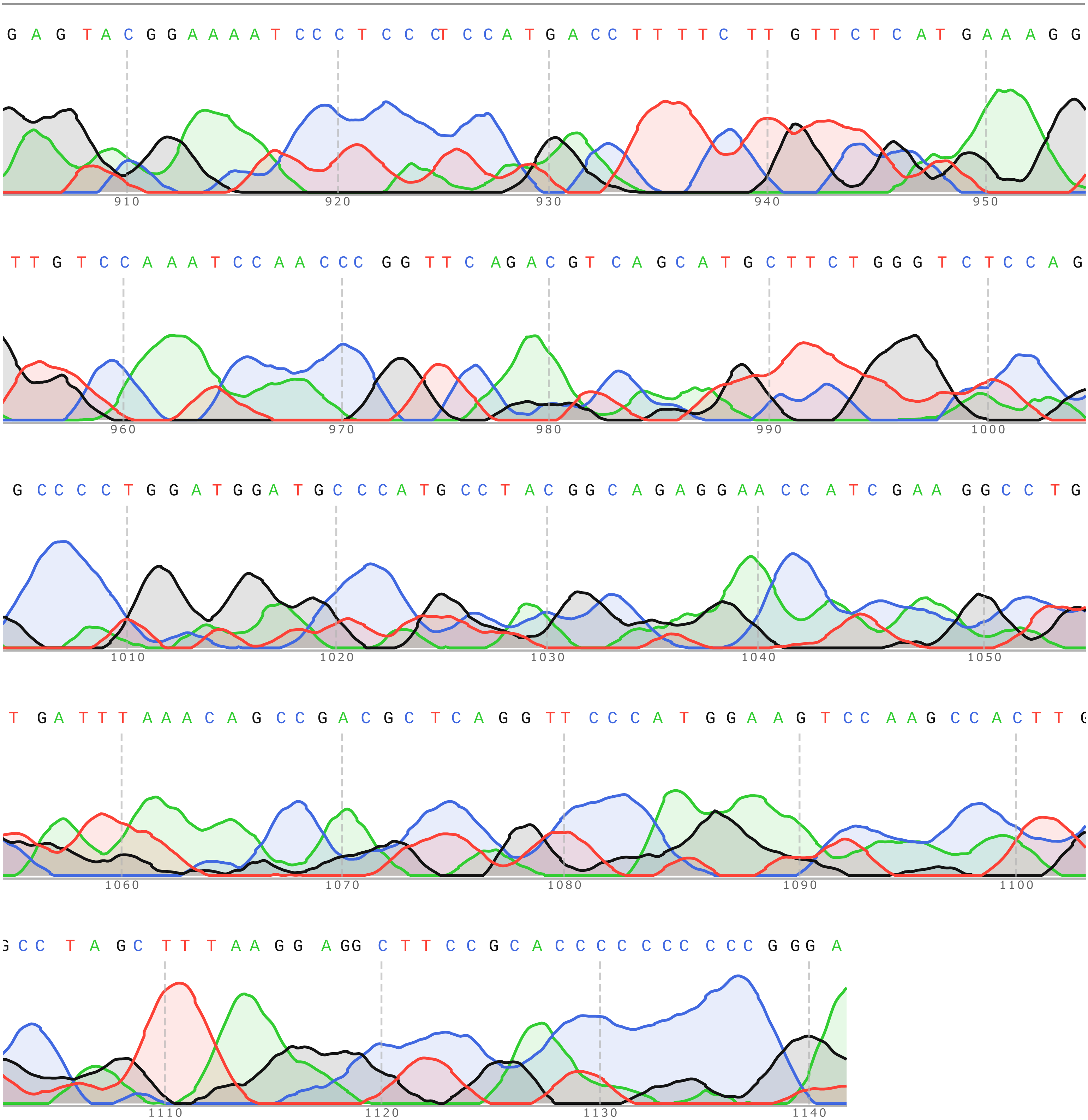
G A G T A C G G A A A A T C C C T C C CT C C A T G A C C T T T T C T T G T T C T C A T G A A A G G
910 920 930 940 950
T T G T C C A A A T C C A A C C C G G T T C A G A C G T C A G C A T G C T T C T G G G T C T C C A G
960 970 980 990 1000
G C C C C T G G A T G G A T G C C C A T G C C T A C G G C A G A G G A A C C A T C G A A G G C C T G
1010 1020 1030 1040 1050
T G A T T T A A A C A G C C G A C G C T C A G G T T C C C A T G G A A G T C C A A G C C A C T T
1060 1070 1080 1090 1100
C C T A G C T T T A A G G A G G C T T C C G C A C C C C C C C C C C G G G A
1110 1120 1130 1140
